# Transferrin Receptor‐Mediated Iron Uptake Promotes Colon Tumorigenesis

**DOI:** 10.1002/advs.202207693

**Published:** 2023-01-26

**Authors:** Hyeoncheol Kim, Luke B Villareal, Zhaoli Liu, Mohammad Haneef, Daniel M Falcon, David R Martin, Ho‐Joon Lee, Michael K Dame, Durga Attili, Ying Chen, James Varani, Jason R. Spence, Olga Kovbasnjuk, Justin A Colacino, Costas A. Lyssiotis, Henry C Lin, Yatrik M Shah, Xiang Xue

**Affiliations:** ^1^ Department of Biochemistry and Molecular Biology University of New Mexico Albuquerque NM 87131 USA; ^2^ Department of Pathology University of New Mexico Albuquerque NM 87131 USA; ^3^ Department of Molecular and Integrative Physiology University of Michigan Ann Arbor MI 48109 USA; ^4^ Department of Internal Medicine Division of Gastroenterology University of Michigan Ann Arbor MI 48109 USA; ^5^ Department of Pathology The University of Michigan Medical School Ann Arbor MI 48109 USA; ^6^ Center for clinical research and translational medicine Yangpu hospital Tongji University School of Medicine Shanghai 200090 China; ^7^ Division of Gastroenterology and Hepatology Department of Medicine the University of New Mexico Albuquerque NM 87131 USA; ^8^ Department of Environmental Health Sciences University of Michigan Ann Arbor MI 48109 USA; ^9^ Section of Gastroenterology Medicine Service New Mexico VA Health Care System Albuquerque NM 87108 USA

**Keywords:** colon, DNA damage response, iron, TFRC, *β*‐catenin

## Abstract

Transferrin receptor (TFRC) is the major mediator for iron entry into a cell. Under excessive iron conditions, TFRC is expected to be reduced to lower iron uptake and toxicity. However, the mechanism whereby TFRC expression is maintained at high levels in iron‐enriched cancer cells and the contribution of TFRC to cancer development are enigmatic. Here the work shows TFRC is induced by adenomatous polyposis coli (APC) gene loss‐driven *β*‐catenin activation in colorectal cancer, whereas TFRC‐mediated intratumoral iron accumulation potentiates *β*‐catenin signaling by directly enhancing the activity of tankyrase. Disruption of TFRC leads to a reduction of colonic iron levels and iron‐dependent tankyrase activity, which caused stabilization of axis inhibition protein 2 (AXIN2) and subsequent repression of the *β*‐catenin/c‐Myc/E2F Transcription Factor 1/DNA polymerase delta1 (POLD1) axis. POLD1 knockdown, iron chelation, and TFRC disruption increase DNA replication stress, DNA damage response, apoptosis, and reduce colon tumor growth. Importantly, a combination of iron chelators and DNA damaging agents increases DNA damage response and reduces colon tumor cell growth. TFRC‐mediated iron import is at the center of a novel feed‐forward loop that facilitates colonic epithelial cell survival. This discovery may provide novel strategies for colorectal cancer therapy.

## Graphical abstract

Transferrin receptor (TFRC)‐mediated iron uptake in colon tumors is essential to maintain the activity of tankyrase (TNKS). TFRC deficiency results in low iron status, causing decreased TNKS activity, stabilized axis inhibition protein 2 (AXIN2), and reduced polymerase delta 1 (POLD1) expression. POLD1 has emerged as a key protein in genome maintenance, and POLD1 deficiency leads to replicative stress.

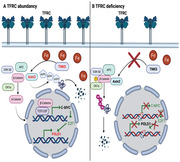

## 1 Introduction

Colorectal cancer (CRC) is the third most prevalent cancer and the third leading cause of cancer‐related deaths in the USA.^[^ ^1^ ^]^ It is projected that the global CRC incidence will become the most common cancer by 2040.^[^ ^2^ ^]^ Thus, there is a great need for mechanistic studies into CRC to propel the development of effective treatments. Consumption of red and processed meats has been associated with a significant risk of developing CRC.^[^ ^3^ ^]^ Red meat is enriched with heme iron, predominantly from hemoglobin and myoglobin, which can be directly absorbed by the small intestine.^[^ ^4^ ^]^ Strikingly, tumor load was almost doubled in the intestine tumor‐prone adenomatous polyposis coli (*Apc)* ^Min/+^ mice fed with a 2.5% hemoglobin diet for 49 days, indicating heme iron is a potent driver of colon tumorigenesis.^[^ ^5^ ^]^ Although previous work has demonstrated that micronutrient iron plays a major role in colon tumorigenesis, the underlying mechanism whereby iron regulates tumor growth is not completely understood.

The expression patterns for many iron‐related genes are altered significantly in CRC tissues relative to normal colon tissues.^[^ ^6^ ^]^ We have demonstrated that the major apical dietary iron uptake transporter divalent metal transporter 1 (DMT1) is highly increased by hypoxia inducible factor (HIF)‐2*α* in colon tumors.^[^ ^7^ ^]^ Pharmacological inhibition or genetic disruption of colonic DMT1 decreases colon tumorigenesis via the signal transducer and activator of transcription 3 (STAT3) signaling.^[^ ^8^ ^]^ Recently, we found colon‐specific deletion of hepcidin, a systemic iron regulating hormone produced mainly in the liver, decreased intratumoral iron and colon tumor incidence through impairing the nucleotide pool.^[^ ^9^ ^]^ In contrast, colon‐specific deletion of the iron exporter ferroportin caused iron accumulation and increased colon tumorigenesis.^[^ ^9^ ^]^ Thus, in CRC the hepcidin‐ferroportin export machinery in the tumor epithelium, together with increased tumor DMT1‐mediated iron uptake, reduces iron efflux and sequesters iron to maintain the nucleotide pool and sustain proliferation. However, colonic DMT1 deletion does not alter normal intestinal epithelial homeostasis.^[^ ^8^ ^]^ Therefore, there is an additional transport mechanism that regulates iron uptake for epithelial cells. Moreover, DMT1 cannot fully explain iron import mechanisms in tumors, as in the absence of DMT1, tumors are reduced but can still grow. Thus, we need to explore more targets to alter iron for better cancer therapy.

Transferrin receptor (TFRC) is the major iron uptake protein on the cell surface that delivers iron into cells through receptor‐mediated endocytosis of di‐ferric transferrin to maintain intracellular iron homeostasis.^[^ ^10^ ^]^ TFRC is absent on the microvilli of villous enterocytes both in normal human and rat duodenal samples.^[^ ^11^, ^12^ ^]^ Instead, TFRC is in the basolateral area of the enterocytes, suggesting that TFRC may transfer systemic iron, but not dietary iron, into enterocytes.^[^ ^13^ ^]^ Moreover, TFRC expression is higher in many types of cancer cells, including CRC cells, than in normal cells, making TFRC is a promising molecular target for diagnosis and treatment of cancer.^[^ ^14^ ^]^

In the present study, we tested the central hypothesis that TFRC‐mediated intratumoral iron accumulation is essential for maintaining nucleotide biosynthesis, DNA damage repair and cell survival in CRC. We found that high expression of TFRC increased iron uptake and accumulation in CRC, whereas TFRC disruption caused iron reduction in CRC. Transcriptomics analysis identified that iron chelation reduced iron‐sulfur protein DNA polymerase delta 1 (POLD1). Mechanistic study revealed that iron was required for the activity of tankyrase (TNKS), which can cause degradation of Axin2 and activate *β*‐catenin signaling.^[^ ^15^ ^]^ In contrast, iron chelation caused downregulation of *β*‐catenin target gene c‐Myc, which subsequently reduced the transcription factor E2F1 and its target gene POLD1. Like iron chelation and TFRC disruption, POLD1 reduction caused increased DNA replication stress, DNA damage response (DDR), apoptosis and repressed tumor growth. Importantly, a combinational effect was found between iron restriction and inhibition of DNA damage signaling proteins, providing a desirable strategy for CRC treatment.

## 2 Results

### 2.1 TFRC is Increased in Human CRC

**Figure 1:**
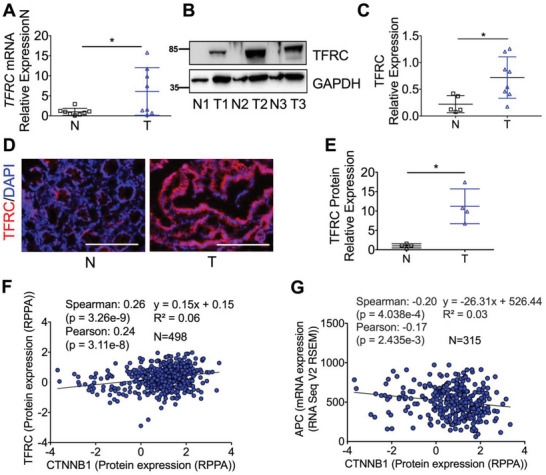
TFRC is increased in human CRC and is positively correlated with the CTNNB1 protein expression. Detection of TFRC expression in human normal (*n* = 5–8) and tumor (*n* = 8) colon tissues by A) qPCR analysis, B) immunoblotting analysis and (C) quantification, and D) immunofluorescence staining and E) quantification. F,G) Correlation analysis using TCGA colorectal adenocarcinoma Firehose legacy dataset from cBioPortal platform. Scale bar = 200 µm. *p < 0.05 and **p < 0.01. Student *t*‐test.

By analyzing the Human Protein Atlas (HPA) database,^[^ ^16^ ^]^ TFRC was expressed in the epithelial cells from the normal human colon tissues (Figure S1A, Supporting Information). In colon tumors, TFRC was expressed both in the cytoplasm and basolateral membrane of epithelial cells (Figure S1B, Supporting Information). Based on UALCAN analysis,^[^ ^17^ ^]^ the mRNA and protein expressions of TFRC from The Cancer Genome Atlas (TCGA) and the Clinical Proteomic Tumor Analysis Consortium (CPTAC) datasets are increased in all stages of colon tumors as compared to normal colons (Figure S1C–S1F, Supporting Information), indicating that TFRC upregulation is an early event during colon tumorigenesis. By qPCR (**Figure** **1**A), immunoblotting (Figure 1B,C), and immunofluorescence staining (Figure 1D,E), we confirmed that TFRC expression was relatively low in normal colons, but greatly increased in colon tumors. Using TCGA colorectal adenocarcinoma Firehose legacy dataset from cBioPortal platform, we found that the TFRC protein expression was positively correlated with the CTNNB1 protein expression (Figure 1F), whereas the CTNNB1 protein expression was negatively correlated with APC mRNA expression (Figure 1G). The data further indicate that loss of APC via genetic mutation may activate beta‐catenin/TFRC signaling pathway.

### 2.2 TFRC Disruption Leads to Colon Injury But Does Not Increase Susceptibility to Colitis

To investigate the role of TFRC in colon tissue, CDX2^ERT2^ *Tfrc* ^F/F^ mice were generated to specifically disrupt TFRC in the colon epithelial cells. The body weights and colon lengths were not changed in CDX2^ERT2^ *Tfrc* ^F/F^ mice after tamoxifen (TAM) treatment to induce Cre recombinase compared to *Tfrc* ^F/F^ mice. However, TFRC disruption caused a “cobblestone appearance” in the colon under a dissection microscope, indicating tissue damage exists (Figure S2A, Supporting Information). Immunoblotting analysis confirmed the reduction of TFRC in CDX2^ERT2^ *Tfrc* ^F/F^ mice (Figure S2B, Supporting Information). Hematoxylin and eosin (H&E) staining showed that the colon injury was increased in CDX2^ERT2^ *Tfrc* ^F/F^ mice compared to *Tfrc* ^F/F^ mice (Figure S2C, S2D, Supporting Information). IF staining showed that the cell proliferation marker Ki67 was not changed (Figure S2C, S2E, Supporting Information), but the apoptosis marker cleaved caspase 3 (CC3) was significantly increased in CDX2^ERT2^ *Tfrc* ^F/F^ mice (Figure S2C, S2F, Supporting Information). However, FITC‐dextran assay indicates gut permeability was not changed (Figure S2G, Supporting Information). To examine the impact of colon injury resulting from TFRC disruption under inflammatory conditions, we treated CDX2^ERT2^ *Tfrc* ^F/F^ mice and *Tfrc* ^F/F^ mice with dextran sodium sulfate (DSS) to induce colitis. We found that the body weights and colon lengths were not different between these two groups (Figure S2H, S2I, Supporting Information). Together, these data indicate that TFRC‐mediated iron uptake is imperative to colon cell survival.

### 2.3 TFRC Disruption Prolongs Survival in a Mouse Model of Colon Dysplasia

**Figure 2:**
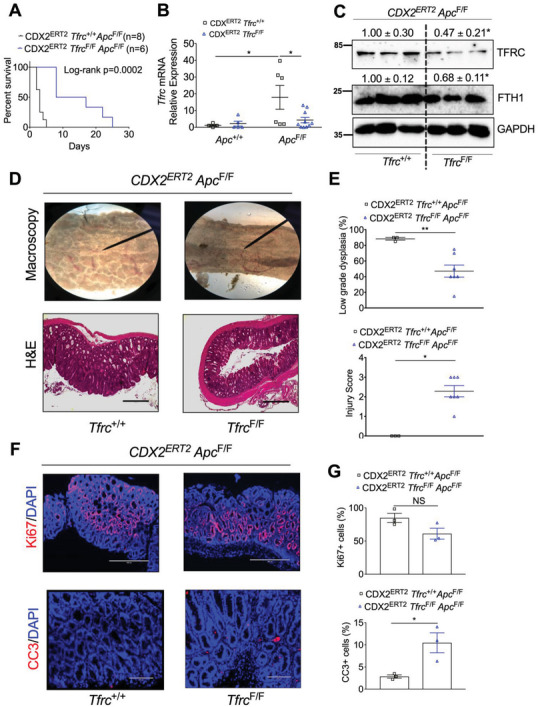
TFRC disruption prolongs mouse survival and reduces low‐grade dysplasia caused by biallelic *Apc* loss. A) Survival curve in *CDX2^ERT2^ Tfrc* ^F/F^ *Apc* ^F/F^ mice (*n* = 8) and *CDX2^ERT2^ Tfrc* ^+/+^ *Apc* ^F/F^ mice (*n* = 6) treated with 100 mg kg^−1^ TAM for 3 days and 1.5% DSS for 7 days. B) qPCR analysis, C) immunoblotting analysis, D) macroscopic and H&E staining images, E) histological scores, F) IF staining, and G) quantification of colons from *CDX2^ERT2^ Tfrc* ^F/F^ *Apc* ^F/F^ mice (*n* = 3–10) and control mice (*n* = 3–6) treated with 100 mg kg^−1^ TAM for 3 days and euthanized 7 days later. Scale bar = 200 µm (CC3 staining) or 400 µm (HE and Ki67 staining). *p < 0.05 and **p < 0.01. NS, not significant. Values above blots are mean ± S.D.

The *APC* gene is mutated in more than 80% of CRC patients.^[^ ^18^ ^]^ To investigate the role of TFRC in colon tumorigenesis, we generated *CDX2^ERT2^ Tfrc* ^F/F^ *Apc* ^F/F^ mice and *CDX2^ERT2^ Tfrc* ^+/+^ *Apc* ^F/F^ mice. After TAM injection and DSS treatment, none of the CDX2^ERT2^ *Tfrc* ^+/+^ *Apc* ^F/F^ mice (*n* = 8) lived beyond 5 days, while all CDX2^ERT2^ *Tfrc* ^F/F^ *Apc* ^F/F^ mice (*n* = 6) did (**Figure** **2**A). DSS‐induced inflammation can accelerate the mouse death and shorten our experimental length, but inflammation also complicates the analysis on gene expression. Thus, we treated another batch of mice with only TAM and euthanized them at day 7 before any death happened. qPCR analysis confirmed that *Apc* depletion increased *Tfrc* mRNA expression, which was blunted by *Tfrc* disruption (Figure 2B). Immunoblot analysis demonstrated that TFRC and the cellular iron storage protein FTH1 were significantly reduced in the colons from CDX2^ERT2^ *Tfrc* ^F/F^ *Apc* ^F/F^ mice compared to *CDX2^ERT2^ Tfrc* ^+/+^ *Apc* ^F/F^ mice (Figure 2C), suggesting other cell types also express TFRC in the mouse colon tissues. Macroscopic and histological analyses indicated that TFRC disruption consistently caused more severe colon injury, but less low‐grade dysplasia (Figure 2D,E). Immunofluorescence staining showed that TFRC depletion increased apoptosis but had no effect on cell proliferation (Figure 2F,G). We hypothesized that this TFRC depletion‐induced apoptosis was due to reduced iron. Indeed, Perl's iron staining demonstrated that the colonic iron deposit was decreased in *CDX2^ERT2^ Tfrc* ^F/F^ *Apc* ^F/F^ mice (Figure S3A, Supporting Information). Iron deficiency increases cell apoptosis by suppressing mTORC1 signaling.^[^ ^19^ ^]^ Indeed, we found that mTORC1 signaling indicated by p‐S6 and p‐S6K levels was significantly reduced in colons from *CDX2^ERT2^ Tfrc* ^F/F^ *Apc* ^F/F^ mice (Figure S3B, Supporting Information). mTORC1 activation is essential for *Apc* deletion‐mediated colon tumorigenesis.^[^ ^20^ ^]^ However, the mTORC1 inhibitor rapamycin did not significantly improve survival rate in *CDX2^ERT2^ Apc* ^F/F^ mice (Figure S3C, Supporting Information). Together, these data show that TFRC knockout causes reduced iron accumulation, increased apoptosis, less dysplasia, and prolonged survival in this biallelic *Apc* loss‐driven dysplasia.

### 2.4 TFRC Depletion Reduces Colon Tumorigenesis by Decreasing Intratumoral iron

**Figure 3:**
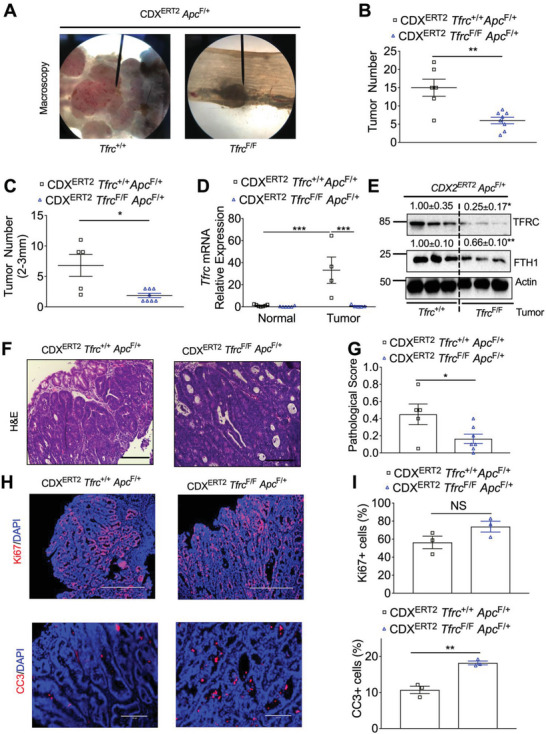
TFRC depletion reduces colon tumorigenesis. A) Macroscopic images, B) total tumor number, C) tumor number at sizes of 2–3 mm, D) qPCR analysis, E) immunoblotting analysis, F–I) histological staining and quantification of colons from *CDX2^ERT2^ Tfrc* ^F/F^ *Apc* ^F/+^ mice (*n* = 3–9) and *CDX2^ERT2^ Tfrc* ^+/+^ *Apc* ^F/+^ mice (*n* = 3–7) treated with TAM (100 mg kg^−1^) for 3 days and 2 cycles of DSS (2%) for 7 days with an interval of 14 days regular water. Scale bar = 200 µm (HE and CC3 staining) or 400 µm (Ki67 staining). *p < 0.05, **p < 0.01, and ***p < 0.001. NS, not significant. Values above blots are mean ± S.D.

As described above, biallelic *Apc* deletion leads to colon dysplasia. We further generated mice with monoallelic *Apc* deletion: CDX2^ERT2^ *Tfrc* ^F/F^ *Apc* ^F/+^ and CDX*2^ERT2^ Tfrc* ^+/+^ *Apc* ^F/+^ mice. After TAM and 2 cycles of DSS treatment, macroscopically visible colon tumors were developed (**Figure** **3**A). TFRC depletion significantly decreased total tumor number (Figure 3B) and the tumor number at sizes of 2–3 mm (Figure 3C). qPCR analysis demonstrated that TFRC was significantly increased in the colon tumors compared to normal colons from CDX*2^ERT2^ Tfrc* ^+/+^ *Apc* ^F/+^ mice, whereas TFRC disruption greatly reduced the increased TFRC in colon tumors from CDX*2^ERT2^ Tfrc* ^F/F^ *Apc* ^F/+^ mice (Figure 3D). Immunoblotting analysis showed that TFRC and FTH1 protein expression were reduced in colon tumors from CDX2^ERT2^ *Tfrc* ^F/F^ *Apc* ^F/+^ mice (Figure 3E). Histological analysis found the tumor pathological score was significantly decreased in CDX*2^ERT2^ Tfrc* ^+/+^ *Apc* ^F/+^ mice compared to CDX*2^ERT2^ Tfrc* ^F/F^ *Apc* ^F/+^ mice (Figure 3F,G). Similarly, Immunofluorescence staining showed that TFRC depletion increased apoptosis but not cell proliferation (Figure 3H,I). Together, these results indicate that TFRC depletion reduces colon tumorigenesis.

To investigate the impact of iron on TFRC expression in normal colon tissues, we treated wildtype C57BL/6 mice with diets containing different amounts of iron. We found that iron diets dose‐dependently reduced TFRC protein expression, but induced FTH1 protein expression in the colon tissues (Figure S4A, Supporting Information), indicating our diets are effective in modulating colonic iron levels. However, high iron diet (1000 mg kg^−1^ ferric citrate iron, 1000 Fe) only significantly reduced TFRC protein, but did not increase FTH1 protein in the liver tissues from wildtype C57BL/6 mice (Figure S4B, Supporting Information), indicating a tissue‐specific response. To further understand the effect of iron on TFRC in colon tumors, we utilized tissues from CDX*2^ERT2^ Apc* ^F/+^ mice that were treated with 1.5% DSS and a high iron diet or an iron‐replete diet (40 mg kg^−1^ iron, 40Fe).^[^ ^8^ ^]^ TFRC was significantly increased in colon tumors compared to normal colons from 40Fe treated mice (Figure S4C–S4E, Supporting Information). Surprisingly, the induction of TFRC in colon tumors was further potentiated by the high iron diet (Figure S4C–S4E, Supporting Information). The expression of FTH1 in normal colon tissues was induced by 1000Fe diet (Figure S4D, Supporting Information), indicating the effectiveness of our diet treatment. To further investigate whether TFRC is critical for high iron‐driven colon tumorigenesis, we treated CDX2^ERT2^ *Tfrc* ^F/F^ *Apc* ^F/+^ and CDX*2^ERT2^ Tfrc* ^+/+^ *Apc* ^F/+^ mice with an iron‐replete diet or a high iron diet. We found that TFRC depletion significantly decreased total tumor number (Figure S4F, Supporting Information) and tumor burden under both diets (Figure S4G, Supporting Information). The tumor numbers at sizes of 1–2 and 2–3 mm were also reduced by TFRC depletion in mice treated with a high iron diet (Figure S4H, Supporting Information). Together, these results indicate that TFRC depletion is effective in reducing iron‐driven colon tumorigenesis.

### 2.5 The DNA Polymerase POLD1 is Regulated by Iron/TNKS/Axin2/*β*‐catenin/c‐Myc/E2F1 Axis

**Figure 4:**
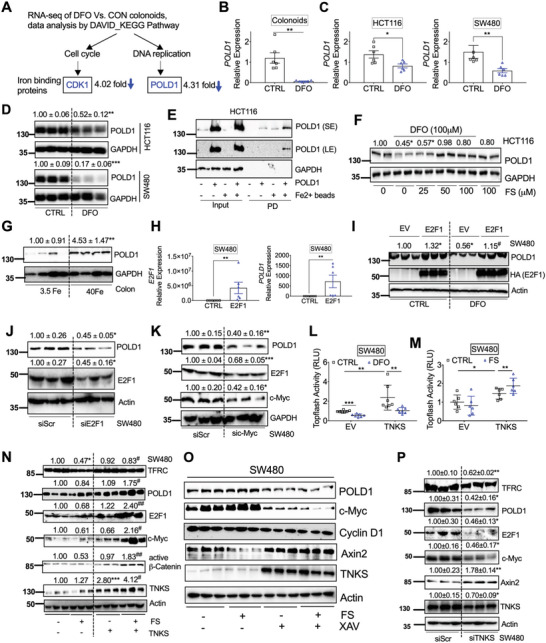
The DNA polymerase POLD1 is regulated by iron/TNKS/Axin2/c‐Myc/E2F1 axis. A) RNA‐seq analysis followed by DAVID bioinformatics analysis and KEGG pathway enrichment identified decreased expression of the iron binding proteins CDK1 in the cell cycle pathway and POLD1 in the DNA replication pathway after DFO treatment in colonoids. Tumor colonoids were treated with DFO (0 or 100 µm) in KGMG medium for 4 days. qPCR analysis of mRNA expression of POLD1 in (B) colonoids, C) HCT116 and SW480 after DFO treatment. D) Immunoblotting blot analysis in colon derived HCT116 or SW480 cancer cells following DFO (100 µm) treatment. E) Immunoblotting analysis of proteins pulled down (PD) by Fe^2+^ or empty beads in HCT116 cells. F) Immunoblotting analysis of HCT116 cells treated with or without DFO (100 µm) and/or different doses of FS. G) Immunoblotting analysis of POLD1 expression in colons from C57BL/6 mice (*n* = 4) treated with 3.5 or 40 Fe. H) qPCR analysis of SW480 cells transfected with E2F1. I) Immunoblotting analysis of SW480 cells transfected with E2F1 and treated with or without DFO (100 µm). Immunoblotting analysis in SW480 cells transfected with J) siE2F1, K) siMYC or a scrambled control (siScr) for 24 h. Topflash luciferase assay in SW480 cells transfected with TNKS or empty vector (EV) for 24 h and then treated with L) DFO (100 µm) or M) FS (100 µm) for an additional 24 h. N) Immunoblotting analysis of SW480 cells transfected with TNKS for 24 h and then treated with FS (100 µm) for overnight. O) Immunoblotting analysis of SW480 cells treated with FS (100 µm) and TNKS inhibitor XAV939 (10 µm) for overnight. P) Immunoblotting blot analysis in SW480 transfected siScr or TNKS siRNA. *p < 0.05, **p < 0.01, and ***p < 0.001 compared with untreated control (CTRL), 3.5 Fe or siScr. #*p* < 0.05 and ##*p* < 0.01 compared with DFO or FS. Values above blots are either mean or mean ± S.D.

To gain insight into how TFRC‐mediated iron reduction affects human colon tumorigenesis, we treated patient‐derived tumor colonoids with iron chelator deferoxamine (DFO) and performed an RNA‐seq analysis. Using differential gene expression analysis followed by the Database for Annotation, Visualization, and Integrated Discovery (DAVID) functional annotation and Kyoto Encyclopedia of Genes and Genomes (KEGG) pathway enrichment,^[^ ^21^ ^]^ we discovered that the cell cycle and DNA replication pathways were enriched in DFO‐treated colonoids (**Figure** **4**A; and Tables S1 and S2, Supporting Information). Using previously identified iron‐interacting proteins to compare the individual genes in these two pathways,^[^ ^8^ ^]^ we noticed that the gene expression of cyclin dependent kinase 1 (CDK1) and POLD1 were decreased 4.02‐ and 4.31‐fold by DFO in tumor colonoids, respectively (Figure 4A; and Tables S1, S3, and S4, Supporting Information). We have previously revealed the important role of the cell cycle protein CDK1 in iron‐driven colon tumorigenesis,^[^ ^8^ ^]^ whereas POLD1 is a key DNA polymerase that synthesizes DNA from deoxyribonucleoside triphosphate.^[^ ^22^ ^]^ However, it is not known whether POLD1 plays a role in iron‐driven colon tumorigenesis and how iron regulates POLD1 mRNA expression. In this work, we first confirmed by qPCR analysis that DFO treatment significantly reduced the mRNA expression of POLD1 in tumor colonoids (Figure 4B). DFO also decreased the mRNA and protein expressions of POLD1 in colon derived HCT116 and SW480 cells (Figure 4C,D). POLD1 was found in our previous ferrous iron beads binding and proteomics identification assay,^[^ ^8^ ^]^ indicating a direct binding between iron and POLD1. Here we confirmed that POLD1 could directly bind to iron in HCT116 cells by immunoblot analysis (Figure 4E). Moreover, ferrous sulfate (FS) supplementation can rescue DFO decreased POLD1 (Figure 4F). However, iron alone did not induce POLD1 (Figure 4F). Furthermore, a 2 week low iron diet (3.5 ppm Fe) decreased POLD1 in mouse colons (Figure 4G), but a high iron diet did not change the expression of POLD1. DFO is known to activate HIF signaling,^[^ ^23^ ^]^ whereas p53 can repress POLD1.^[^ ^24^ ^]^ However, neither inhibiting HIF signaling nor p53 knockout rescued POLD1 repression by DFO (Figure S5A–S5C, Supporting Information). E2F1 is a POLD1 activating transcription factor and a direct target gene of c‐Myc,^[^ ^25^, ^26^ ^]^ whereas DFO reduces c‐Myc.^[^ ^27^ ^]^ We found that DFO reduced E2F1, c‐Myc, POLD1, and FTH1, and increased the intestinal iron‐responsive protein HIF‐2*α* as we our previously reported^[^ ^7^ ^]^ (Figure S5D, Supporting Information), indicating DFO decreased intracellular iron levels. Importantly, E2F1 overexpression increased POLD1 mRNA (Figure 4H), rescued POLD1 repression by DFO (Figure 4I), whereas E2F1 knockdown (Figure 4J), c‐Myc knockdown (Figure 4K), and c‐Myc inhibitor (c‐Myci) 10058‐F4 (Figure S5E, Supporting Information), reduced E2F1 and POLD1. These results indicate that c‐Myc and E2F1 play a critical role in POLD1 regulation under iron starvation.

c‐Myc is a known *β*‐catenin direct target gene,^[^ ^28^ ^]^ whereas Wnt inhibitor screen reveals *β*‐catenin signaling is iron‐dependent.^[^ ^29^ ^]^ Consistently, we found that iron chelation by DFO significantly reduced basal and *β*‐catenin induced Topflash luciferase activity (Figure S5F, Supporting Information), which is an indicator of *β*‐catenin signaling activity. Iron was reported to amplify Wnt signaling and induce c‐Myc expression in SW480 cells with APC mutation,^[^ ^30^ ^]^ though surprisingly iron did not activate or potentiate Wnt signaling in our experiments.^[^ ^8^ ^]^ Immunoblot analysis confirmed that iron reduced TFRC, Axin2, and the iron‐sensing iron regulatory protein (IRP) 2, and increased FTH1 as expected (Figure S5G, Supporting Information). However, iron treatment did not change c‐Myc, E2F1, POLD1, and another *α*‐catenin target gene, cyclin D1 (Figure S5G, Supporting Information). DFO‐mediated iron starvation response increased TFRC and decreased cyclin D1 as previously reported,^[^ ^31^ ^]^ but it also induced Axin2 (Figure S5H, Supporting Information). Axin2 is a transcriptional target and a negative regulator of *β*‐catenin signaling.^[^ ^32^ ^]^ Consistently, Axin2 knockdown by siRNA induced TFRC, c‐Myc, E2F1, and POLD1 (Figure S5I, Supporting Information). TNKS is a Zn binding protein important in Axin2 poly‐ADP‐ribosylation, ubiquitination and degradation.^[^ ^33^ ^]^ Divalent metals often compete with other divalent metals for protein binding. We found neither iron supplementation nor iron chelation changed TNKS expression (Figure S5J, S5K, Supporting Information). However, iron chelation significantly reduced basal and TNKS‐induced Topflash luciferase activity (Figure 4L), whereas iron supplementation increased TNKS‐induced but not basal Topflash luciferase activity (Figure 4M). These data indicate that iron is required for TNKS activity. Furthermore, the combination of iron supplementation and TNKS overexpression, but neither treatment alone, greatly activated the TNKS/Axin2/*β*‐catenin signaling and induced TFRC, POLD1, E2F1, c‐Myc, and Cyclin D1 (Figure 4N). Iron treatment reduced Axin2, whereas TNKS activity inhibitor rescued the protein expression of Axin2 and decreased the expression of POLD1 and c‐Myc (Figure 4O). Consistently, TNKS knockdown reduced TNKS, c‐Myc, E2F1, and POLD1, but increased Axin2 (Figure 4P). Collectively, these data indicate that iron is essential for TNKS/Axin2/*β*‐catenin/c‐Myc/E2F1/POLD1 signaling.

### 2.6 TFRC Depletion Leads to Decreased Iron Levels, POLD1 Expression, and Tumor Growth

**Figure 5:**
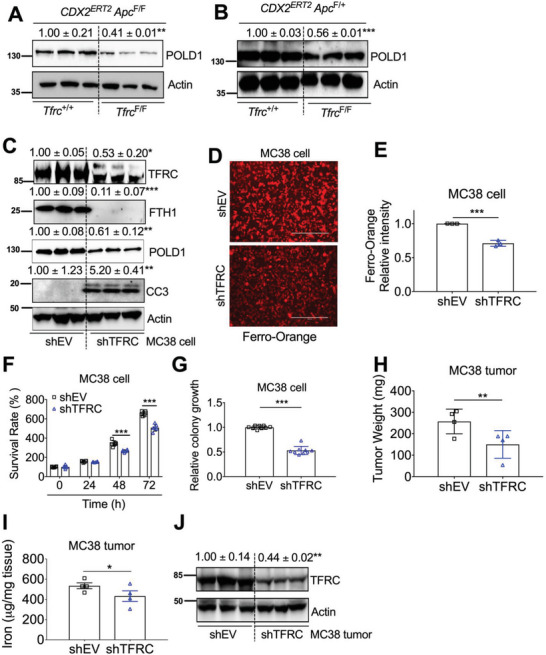
TFRC depletion causes decreased iron, POLD1 and tumor growth. Immunoblotting analysis in (A) dysplastic colons from *CDX2^ERT2^ Tfrc* ^F/F^ *Apc* ^F/F^ and *CDX2^ERT2^ Tfrc* ^+/+^ *Apc* ^F/F^ mice (*n* = 3). B) Colon tumors from *CDX2^ERT2^ Tfrc* ^F/F^ *Apc* ^F/+^ and *CDX2^ERT2^ Tfrc* ^+/+^ *Apc* ^F/+^ mice (*n* = 3). C) Immunoblotting analysis, D) Ferro‐Orange staining and E) quantification, F) MTT assay, G) colony formation assay in MC38 cells with or without TFRC knockdown. H) Tumor weight, I) tumor iron levels, and J) immunoblotting analysis of tumor xenografts from MC38 cells with or without TFRC knockdown (*n* = 3–4). *p < 0.05, **p < 0.01, and ***p < 0.001 compared with *Tfrc* ^+/+^ mice or shEV. Values above blots are mean ± S.D.

Because TFRC knockout leads to decreased iron in colon, and iron starvation can reduce POLD1 expression, we further investigated whether TFRC disruption could reduce POLD1 expression. Indeed, POLD1 was decreased by TFRC disruption (**Figure** **5**A,B). To confirm the TFRC‐mediated change of POLD1 is a direct effect in epithelial cells, TFRC was stably knocked down by shRNA in colon‐derived murine MC38 cells. TFRC, FTH1, POLD1, E2F1, and c‐Myc were decreased, whereas CC3 and Axin2 were increased and TNKS was not changed in MC38 shTFRC cells (Figure 5C; and Figure S6A, Supporting Information). Furthermore, E2F1 overexpression rescued TFRC knockdown mediated POLD1 reduction and CC3 activation (Figure S6B, Supporting Information). To further confirm that TFRC knockdown caused cellular iron status change, we performed Ferro–Orange staining. FS greatly increased, whereas DFO greatly reduced, the orange fluorescence signal in MC38 cells (Figure S6C, Supporting Information), indicating that this dye is a good probe for cellular ferrous iron levels. Thus, the decreased fluorescence intensity in MC38 shTFRC cells indicates reduced cellular ferrous iron levels (Figure 5D,E). Both MTT assay and colony formation assay showed that MC38 shTFRC cells exhibited reduced cell growth (Figure 5F,G; and Figure S6D, Supporting Information). Furthermore, MC38 shTFRC cell‐derived tumor xenografts had lower weights (Figure 5H). Decreased tumor tissue iron and TFRC were also observed (Figure 5I,J). Together, our data reveal that TFRC depletion in colon epithelial cells can lead to decreased cellular iron levels, reduced POLD1 expression, increased apoptosis, and repressed tumor growth.

### 2.7 POLD1 Inhibition Causes Increased DNA Replicative Stress and Impaired Tumor Growth

**Figure 6:**
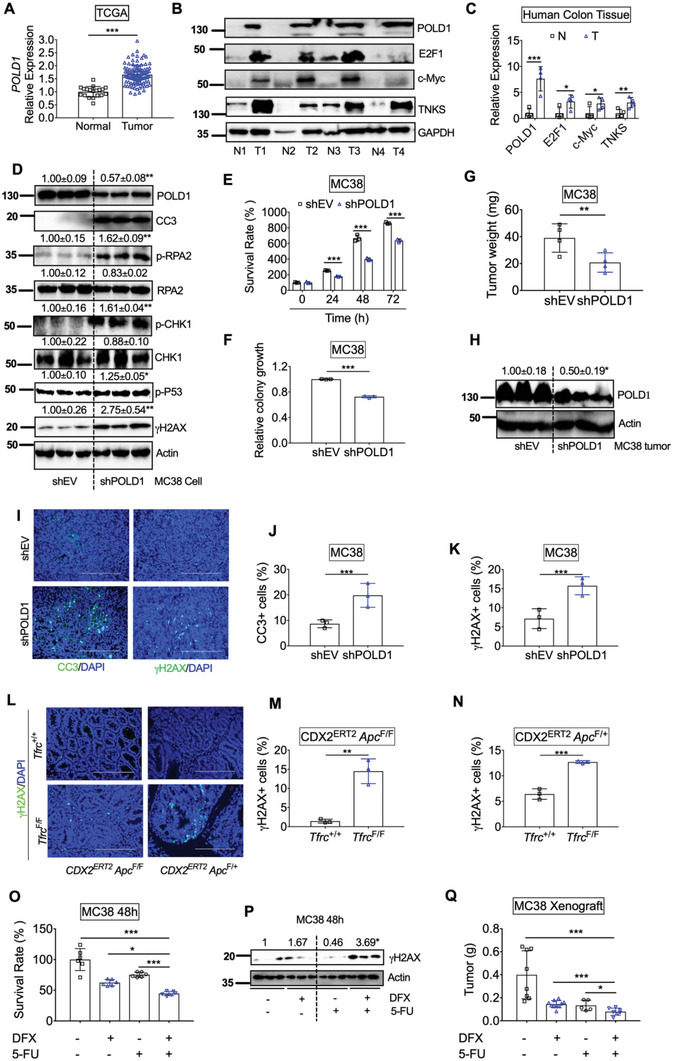
POLD1 inhibition leads to increased DNA replication stress and impaired tumor growth. A) Gene expression of POLD1 in colons from TCGA database. B) Immunoblotting analysis and C) quantification of protein expression in human normal and tumor colons (*n* = 4). D) Immunoblotting analysis, E) MTT assay and F) colony formation assay in MC38 cells with or without stable POLD1 knockdown. G) Tumor weight, H) Immunoblotting analysis, I) immunofluorescence staining, quantification of J) CC3 and K) *γ*H2AX staining in tumor xenografts from MC38 cells with or without POLD1 knockdown (*n* = 3). L) *γ*H2AX staining and quantification of M) dysplastic colon tissues from *CDX2^ERT2^ Tfrc* ^F/F^ *Apc* ^F/F^ and *CDX2^ERT2^ Tfrc* ^+/+^ *Apc* ^F/F^ mice (*n* = 3) and N) colon tumor tissues from *CDX2^ERT2^ Tfrc* ^F/F^ *Apc* ^F/+^ and *CDX2^ERT2^ Tfrc* ^+/+^ *Apc* ^F/+^ mice (*n* = 3). O) MTT assay and P) Immunoblotting analysis in MC38 cells treated with DFX (10 *µ* m) and/or 5‐FU (10 *µ* m) for 48 h. Q) tumor weight for xenografts from MC38 cells treated with DFX and/or 5‐FU (*n* = 5–10). *p < 0.05, **p < 0.01, and ***p < 0.001. NS, not significant. Values above blots are either mean or mean ± S.D.

To further investigate the role of POLD1 in colon tumorigenesis, we first examined POLD1 in human CRC tissues. The mRNA expression of POLD1 was significantly increased in tumors compared to normal colons from TCGA database (**Figure** **6**A). We further confirmed that the protein expression of POLD1 in CRC tumors, together with E2F1, c‐Myc, and TNKS, was increased in a small cohort of paired patient samples (Figure 6B,C). Immunohistochemistry staining from HPA showed that POLD1 was highly increased in colon tumors (Figure S7A, Supporting Information). Besides DNA replication, POLD1 has emerged as a pivotal protein in genome maintenance, and POLD1 deficiency leads to replicative stress.^[^ ^34^ ^]^ DNA replicative stress can occur when oncogenes, genotoxic agents, or inhibitors of DNA replication cause stalled replication forks, leading to activation of DDR pathways. Replicative stress also leads to the phosphorylation of the replication fork component, replication protein A2 (RPA2) to initiate DNA checkpoint signaling.^[^ ^35^ ^]^ Checkpoint kinase 1 (CHK1) is activated by Ataxia Telangiectasia and Rad3‐related protein (ATR) phosphorylation to resolve incomplete DNA replication.^[^ ^36^ ^]^ Unresolved replicative stress can lead to cell death by replication catastrophe, in which exhaustion of replication factors, such as RPA2, triggers widespread DNA double‐strand breaks marked by *γ*‐H2AX.^[^ ^37^ ^]^ Importantly, POLD1 knockdown decreased POLD1, resulting in increased p‐RPA2, p‐CHK1,  *γ*H2AX, p‐p53, and apoptosis marker CC3 in MC38 cells (Figure 6D). Consistently, both MTT assay and colony formation assay showed that MC38 shPOLD1 cells had less cell growth compared to shEV cells (Figure 6E,F). Furthermore, MC38 shPOLD1 cell‐derived tumor xenografts had lower weights (Figure 6G). Immunoblotting confirmed that POLD1 levels were still reduced in xenograft tumors from shPOLD1 cells compared to controls (Figure 6H). Immunofluorescence staining showed that CC3 and *γ*H2AX were significantly increased in xenograft tumors from shPOLD1 cells compared to controls (Figure 6I–K). Notably, *γ*H2AX was also increased in colon dysplastic tissues from *CDX2^ERT2^ Tfrc* ^F/F^ *Apc* ^F/F^ mice and colon tumors from *CDX2^ERT2^ Tfrc* ^F/F^ *Apc* ^F/+^ mice (Figure 6L–N). Consistently, DFO treatment significantly increased p‐RPA2, p‐CHK1, p‐p53, *γ*H2AX, and CC3 in CRC cells (Figure S7B, Supporting Information).

A recent synthetic lethal screen showed that ATR‐ or CHK1‐inhibitors potentiate caspase‐dependent apoptosis in POLD1‐deficient cancers.^[^ ^38^ ^]^ Another study showed that pharmacologic blockade of B‐family DNA polymerases using aphidicolin combined with CHK1 inhibitors also led to synergistic inhibition of cancer cell proliferation.^[^ ^39^ ^]^ However, aphidicolin is too toxic to be used in humans, and currently no other suitable drug candidates are able to block the DNA polymerase family exist. We tested the combinatorial effect of low doses of both DFO and a potent CHK1 inhibitor, UCN‐01, on the survival of CRC cells. We found that the growth inhibition effect in SW480 cells was better in the combination group of 10 µm DFO and 100 nm UCN‐01 than either drug alone (Figure S7C, Supporting Information). This effect was further validated with a more specific CHK1 inhibitor, Prexasertib (Figure S7D, Supporting Information),^[^ ^40^ ^]^ an oral iron chelator, deferasirox (DFX) (Figure S7E, Supporting Information), and an ATR inhibitor, VE‐822 (Figure S7F, Supporting Information). Interestingly, the combination of DFX with Prexasertib or VE‐822 decreased POLD1 and increased *γ*H2AX (Figure S7G–S7J, Supporting Information), which was used as a bioactivity marker for CHK1 inhibitors.^[^ ^41^ ^]^ Importantly, 5‐FU, a standard first‐line DNA damaging chemotherapy drug for CRC, also potentiated the growth‐suppressive effect and DDR of DFX on MC38 cells in vitro and in vivo without affecting mouse body weights (Figure 6O–Q). Collectively, we propose that the combination effect between iron chelation and DDR inhibition may be utilized as a novel therapeutic strategy for CRC.

## 3 Discussion

**Figure 7:**
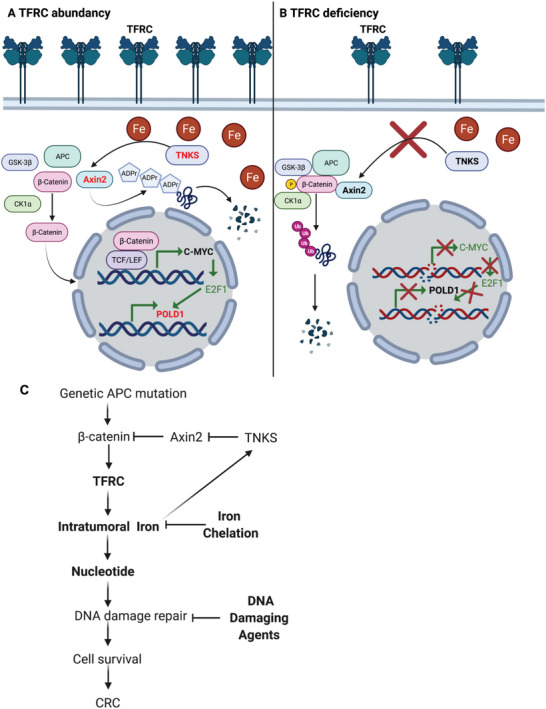
Working model. A) TFRC‐mediated iron uptake in colon tumors is required for maintaining the activity of metal‐dependent TNKS, which causes poly‐ADP‐ribosylation and degradation of Axin2, and activation of *β*‐catenin/c‐Myc/E2F1/POLD1 signaling. B) TFRC deficiency‐caused low iron status leads to decreased TNKS activity, stabilized Axin2, *β*‐catenin phosphorylation and degradation, suppressed c‐Myc/E2F1/POLD1 transcription, increased DNA replicative stress, DNA damage and subsequent cell apoptosis. C) TFRC is induced by active *β*‐catenin signaling due to genetic APC mutation, whereas TFRC‐mediated intratumorally iron accumulation potentiates *β*‐catenin signaling via directly enhancing the activity of TNKS. TFRC‐mediated iron import is at the center of this feed‐forward loop to facilitate tumor cell survival via supplying nucleotides for DNA damage repair in CRC. Combination of iron chelation and DNA damaging agents increases DNA damage and suppresses cell growth in CRC.

Using a novel colon epithelial cell specific TFRC knockout mouse model, we found TFRC disruption impeded colon tumorigenesis by decreasing intratumoral iron accumulation. Mechanistic study revealed that TFRC‐mediated iron uptake in colon tumors is required to maintain the activity of the metal‐dependent TNKS. TNKS activity caused poly‐ADP‐ribosylation and degradation of Axin2 and activation of *β*‐catenin/c‐Myc/E2F1/POLD1 signaling (**Figure** **7**A). TFRC deficiency‐elicited low iron status decreased TNKS activity, stabilized Axin2, increased *β*‐catenin phosphorylation, and degradation, suppressed c‐Myc/E2F1/POLD1 transcription, and increased DNA replicative stress, DNA damage, and cell apoptosis (Figure 7B). Axin2 knockdown increased, whereas TNKS knockdown decreased, the expression of TFRC. Furthermore, overexpression of TNKS potentiated *β*‐catenin signaling in the presence of iron and rescued iron‐repressed TFRC. Thus, we hypothesize that TFRC is induced by active *β*‐catenin signaling, whereas TFRC‐mediated intratumorally iron accumulation potentiates *β*‐catenin signaling by directly enhancing the activity of TNKS (Figure 7C). TFRC‐mediated iron uptake is at the center of this feed‐forward loop, which facilitates tumor cell survival in CRC.

TFRC plays a key role in iron transportation and is also involved in ferroptosis, a new type of regulated cell death driven by the iron‐dependent accumulation of oxidized polyunsaturated fatty acid‐containing phospholipids.^[^ ^42^ ^]^ Unlike apoptosis, ferroptosis is an immunogenic cell death that can trigger leakage of damage‐associated molecular patterns and activate host inflammatory response.^[^ ^43^ ^]^ Ferroptosis is reported to be implicated in inflammatory bowel disease, including ulcerative colitis and Crohn's disease.^[^ ^44^ ^]^ In light of the essential role of TFRC in ferroptosis, it is conceivable that blockade of ferroptosis by loss of TFRC preferentially contributes to the suppression of colitis and colitis‐associated cancer development. However, we observed TFRC disruption caused mild colon injury shown as increased epithelial cell apoptosis but did not change colitis susceptibility. Our data indicates that the stimulatory effects on apoptosis, rather than the blockage of ferroptosis‐related inflammation, contributes to decreased colon tumor growth after TFRC loss.

Excess iron is expected to decrease TFRC to reduce iron uptake and ferroptosis. However, we found that TFRC expression was paradoxically induced in iron‐enriched colon tumors and further potentiated by a high‐iron diet. TFRC was significantly induced in the colons after *Apc* gene disruption and across CRC stages, indicating that tumor cells are dependent upon iron and have developed mechanisms to tolerate iron toxicity. For human colon tumors without *APC* gene mutation, ferritinophagy, and hypoxia signaling pathways may be implicated in the induction of TFRC.^[^ ^45^, ^46^ ^]^ It is surprising that TFRC deletion has no effect on cell proliferation, but only increases apoptosis. This could be because DMT1‐mediated iron trafficking from the apical side and other iron import mechanisms are still functional in mice fed with iron rich diets, thus proliferation is maintained in the TFRC knockout models.

It is also intriguing that iron suppressed Axin2 expression but failed to induce c‐Myc/E2F1/POLD1 axis. However, Axin2 knockdown and TNKS overexpression with iron supplementation activated c‐Myc/E2F1/POLD1 axis. c‐Myc is greatly induced by biallelic *Apc* inactivation in mouse intestines,^[^ ^47^ ^]^ which is completely abolished by monoallelic inactivation of *β*‐catenin encoding gene *Ctnnb1*.^[^ ^48^ ^]^ Proteins from the TNKS/c‐Myc/E2F1/POLD1 axis was also increased in human colon tumors. Thus, it is important to determine what mechanism drives TNKS overexpression in CRC in the future.

POLD1 is an iron‐sulfur cluster containing protein and the iron‐sulfur cluster was incorporated by the cytosolic iron‐sulfur cluster assembly system.^[^ ^49^ ^]^ Thus, iron is known to modulate the stability and activity of POLD1 at the post‐transcriptional level. Our data further showed that reduced iron can also repress POLD1 at the transcriptional level through TNKS/Axin2/*β*‐catenin/c‐Myc/E2F1 signaling. Germline and nonsilent somatic mutations in the exonuclease proofreading domains of POLD1 predispose patients to develop hypermutated sporadic and colitis‐associated CRC.^[^ ^22^ ^]^ We found POLD1 was correlated with c‐Myc. Thus, c‐Myc is the rheostat that connects POLD1 expression with *β*‐catenin signaling in CRC.

Because TFRC is overexpressed in a variety of human tumors, antibodies targeting TFRC are attractive and straightforward therapeutic options.^[^ ^14^ ^]^ However, the safety of this type of treatment is a major concern, as many normal tissues like bone marrow and lymphoid tissues express high levels of TFRC.^[^ ^16^ ^]^ In contrast, the FDA‐approved iron chelator DFO has a relatively safe record in the clinical treatment of iron overload diseases. DFO treatment resulted in 20% overall response rate in a cohort of 10 advanced hepatocellular carcinoma patients without severe adverse events.^[^ ^50^ ^]^ However, DFO's extremely short circulation half‐life restricts its use as an effective antitumor agent.^[^ ^51^ ^]^ When DFO is used in iron overload diseases, the drug must be administered by continuous infusion for up to 12 h per day for 5–7 days per week, which leads to suboptimal patient adherence to therapy.^[^ ^52^ ^]^ DFO's limited ability to cross cell membranes also limits its efficacy.^[^ ^53^ ^]^ Two next generation orally administered iron chelators, deferiprone and DFX, are now in routine clinical use for treating iron overload diseases.^[^ ^54^ ^]^ To reduce potential acute toxicities and maintain its efficacy, DFX has been investigated for combinatorial therapy in breast cancer.^[^ ^14^ ^]^ However, DFX or other iron chelator‐based drug combinations for CRC treatment have not yet been reported. A metal chelator, Tachpyridine, induces G2 cell cycle arrest via ATR‐dependent activation of CHK1, and sensitizes cancer cells to ionizing radiation induced DNA damage, sparing noncancerous cells.^[^ ^55^ ^]^ DNA damage signaling inhibitors mainly target kinases (e.g., ATR, CHK1) that phosphorylate a range of proteins involved in triggering cell‐cycle arrest to enable DNA repair.^[^ ^56^ ^]^ Since the FDA approved the first poly‐ADP‐ribose polymerase inhibitor Olaparib in 2014, the development of novel DDR targets has gained enormous attention.^[^ ^56^ ^]^ However, DDR inhibitor monotherapy often has limited efficacy for cancer treatment.^[^ ^57^ ^]^ Here we have shown that the combinatorial inhibition of POLD1 by iron chelation and DDR by DNA damaging agents increases DNA damage and suppresses CRC cell growth at a very low dose (Figure 7C).

Notably, when dietary iron is increased, other metals such as manganese and the antioxidant activity of manganese superoxide dismutase are decreased,^[^ ^58^ ^]^ which may also impact tumor development. Also, DFO can chelate other metals in addition to iron. Thus, an iron‐specific chelation‐based chemotherapy strategy is worth further investigation. Also, as patients with colon cancer often present with anemia, the utilization of iron chelators needs to be carefully monitored in these patients.

Taken together, we have elucidated one of potentially many important mechanisms affected by iron availability: TFRC‐mediated iron uptake is essential for colon tumorigenesis via modulating TNKS/Axin2/*β*‐catenin/c‐Myc/E2F1/POLD1 axis.

## 4 Experimental Section

### Cell Culture

Human HCT116, SW480, and murine MC38 CRC cells were maintained at 37 °C in 5% CO_2_ and cultured in Dulbecco's modified Eagle medium (DMEM) supplemented with 10% fetal bovine serum (FBS) and 1% penicillin and streptomycin (VWR, Radnor, PA). HCT116 p53+/+ and p53−/− cells were a kind gift from Professor Bert Vogelstein at the Johns Hopkins University. For generating stable cell lines, MC38 cells were transfected with a mouse shTFRC plasmid (TRCN0000375695, Sigma, St. Louis, MO) or a mouse shPOLD1 plasmid (TRCN0000428858, Sigma) and selected with 1 µg mL^−1^ puromycin.

### Human CRC Tissues

For the human CRC tissues and adjacent normal colon tissues, RNAs, proteins, and frozen sections were prepared from banked snap frozen surgical resection tissues present in the UNM Cancer Center Human Tissue Repository & Tissue Analysis Shared Resource. The UNM Institutional Review Board approved this study (#19‐131).

### Animals

All mice were maintained in a standard cage in a light and temperature‐controlled room and were allowed standard chow and water except as indicated. Both male and female mice were used in the study. *Tfrc* floxed (*Tfrc* ^F/F^) mice (Stock No: 02 8177) and mice with the tamoxifen (TAM)‐inducible caudal type homeobox 2 (CDX2) ^ERT2^‐Cre promoter (Stock No: 02 2390) were both purchased from the Jackson lab and crossed to generate colon‐specific TFRC knockout (*CDX2^ERT2^ Tfrc* ^F/F^) mice. For fluorescein isothiocyanate (FITC)‐dextran gut permeability assay, *CDX2^ERT2^ Tfrc* ^F/F^ and *Tfrc* ^F/F^ mice were treated with 100 mg kg^−1^ TAM for 3 and 7 days later were given 600 mg kg^−1^ FITC‐dextran by oral gavage. For colitis study, *CDX2^ERT2^ Tfrc* ^F/F^ and *Tfrc* ^F/F^ mice were treated with 100 mg kg^−1^ TAM for 3 days, and 7 days later were provided with regular chow and 3% DSS water for 7 days. For the survival analysis, *CDX2^ERT2^ Apc* ^F/F^ and *CDX2^ERT2^ Tfrc* ^F/F^ *Apc* ^F/F^ mice were generated and treated with 100 mg kg^−1^ TAM for 3 consecutive days, and 7 days later were treated with 1.5% DSS for 7 days. They were then put back on regular drinking water. For the qPCR and immunoblotting analysis, *CDX2^ERT2^ Apc* ^F/F^ and *CDX2^ERT2^ Tfrc* ^F/F^ *Apc* ^F/F^ mice were treated with 100 mg kg^−1^ TAM for 3 consecutive days and sacked 7 days later. For establishing the CRC model, *CDX2^ERT2^Apc* ^F/+^ and *CDX2^ERT2^ Tfrc* ^F/F^ *Apc* ^F/+^ mice were generated and treated with 2% DSS for 7 days (inflammatory phase), and then were put on regular drinking water for 14 days (recovery phase). One more inflammatory phase and recovery phase were performed. For iron diet treatment, C57BL/6 mice were provided with low‐iron diet (3.5 mg kg^−1^ ferric citrate iron, 3.5 Fe), iron‐replete diet (40 mg kg^−1^ ferric citrate iron, 40Fe) or high‐iron diet (1000 mg kg^−1^ ferric citrate iron, 1000 Fe) from Research Diets (New Brunswick, NJ) for 2 weeks, whereas *CDX2^ERT2^Apc* ^F/+^ and *CDX2^ERT2^ Tfrc* ^F/F^ *Apc* ^F/+^ mice were treated with 40 or 1000 Fe 2 days before the initiation of DSS treatment. For subcutaneous xenograft study, 1 × 10^6^ syngeneic MC38 cells with *Tfrc* or *Pold1* gene knockdown were injected into the flanks of C57BL/6 mice. Two weeks later, mice were sacrificed, and tumors were collected. For combination therapy, when tumors were palpable at 1 week after subcutaneous injection of MC38 cells, C57BL/6 mice were treated with vehicle, 20 mg kg^−1^ DFX by oral gavage every other day, 1 mg kg^−1^ 5‐fluorouracil (5‐FU) by intraperitoneal injection daily, or the combination of DFX every other day and 5‐FU daily.

### Immunoblotting Analysis

Cells and tumor tissues were lysed with radioimmunoprecipitation assay buffer. After incubation, cell extracts were centrifuged and the supernatant was collected for Bradford assay to quantify protein concentration by a BioTek Synergy HTX Multi‐Mode Microplate Reader (BioTek, Winooski, VT). Equal amounts (20–50 µg) of protein were loaded for sodium dodecyl sulfate polyacrylamide gel electrophoresis (SDS‐PAGE). The proteins inside the gels were transferred onto nitrocellulose membrane with a wet transfer method. The membranes were blocked with 3% milk for 1 h. Primary antibodies were incubated overnight. Secondary antibodies were incubated for 1 h. Antibodies for ferritin heavy chain (FTH1, #3998), active beta catenin (#8814), Anti‐rabbit IgG HRP‐linked Antibody (#7074), and Anti‐mouse IgG HRP‐linked Antibody (#7076) were from Cell signaling Technology (Danvers, MA). Primary antibodies for TFRC (sc‐393719), HIF‐1*α* (sc‐13515), HIF‐2*α* (sc‐13596), pS6 (sc‐514033), pS6K (sc‐8418), p‐p53 (sc‐377567), p53 (sc‐6243), c‐MYC (sc‐40), GAPDH (sc‐47724), Cyclin D1 (sc‐718), and Actin (sc‐8432) were from Santa Cruz Biotechnology (Dallas, TX). P‐RPA2 (PA5‐39809) was from Invitrogen (Carlsbad, CA). POLD1 (15646‐1‐AP), p‐CHK1 (28805‐1‐AP), E2F1 (66515‐1‐Ig), Axin2 (20540‐1‐AP), and TNKS (18030‐1‐AP) were from Proteintech (Rosemont, IL).

### Patient‐Derived Colorectal Tumor Colonoids

The adenoma colonoids culture was described previously.^[^ ^8^ ^]^ These human colonoids contain mutations in APC Thr1556fs, KRAS Ala146Val, MET Arg988Cys, PMS2 Gly29Ala, TP53 Arg267Trp, EP300 Asp1579Asn. The culture plates were placed in a 37 °C incubator for 30 min to solidify the Matrigel, followed by the addition of 1 mL serum‐free Keratinocyte Growth Media Gold (KGMG, Catalogue: 001 95769, Lonza, Basel, Switzerland). After 24 h of plating, colonoids were treated with DFO (0 or 100 µm) in KGMG for 4 days with fresh media change every other day. At the end of the experiment, culture media was aspirated off, and colonoids were lysed with Trizol to extract RNA.

### Statistical Analysis

Descriptive results were expressed as mean ± standard deviation. Unless otherwise specified, all in vitro assays were performed in biological triplicates and independently repeated three times to confirm reproducibility of the results. *p* values were calculated by independent *t*‐test, paired *t*‐test, one‐way, and two‐way analysis of variance (ANOVA). *p* < 0.05 was considered significant.

### Study Approval

Animal studies were performed in accordance with the Institute of Laboratory Animal Resources guidelines and approved by the Institutional Animal Care and Use Committee (IACUC) at the University of New Mexico Health Sciences Center (Protocol# HSC‐18‐200699, 20‐201060‐HSC) and followed the National Institutes of Health guide for the care and use of Laboratory animals (NIH Publications No. 8023, revised 1978). The human tissues were obtained from UNM Human Tissue Repository with an approved protocol (#19‐131) by the UNM Health Sciences Institutional Review Board – IRB (aka Human Research Review Committee – HRRC) and any information for the donors is blinded.

## Conflict of Interest

C.A.L. has received consulting fees from Astellas Pharmaceuticals and Odyssey Therapeutics and is an inventor on patents pertaining to Kras regulated metabolic pathways, redox control pathways in cancer, and targeting the GOT1‐pathway as a therapeutic approach.

## Author Contributions

H.K., X.X. designed the study. H.K., L.B.V., L.Z., M.H., D.M.F., D.R.M., H.L., M.K.D., D.A., Y.C., J.A.C., and X.X. performed experiments and analyzed the data. J.V., J.R.S., O.K., C.A.L., H.C.L., Y.M.S., and X.X. planned the project and supervised the experiment. H.K. and X.X. wrote the manuscript.

## Supporting information

Supporting Information

Supplemental TableS1

Supplemental TableS2

Supplemental TableS3

Supplemental TableS4

## Data Availability

The data that support the findings of this study are available from the corresponding author upon reasonable request.
