# Supplementary material for: Transferrin Receptor‐Mediated Iron Uptake Promotes Colon Tumorigenesis
Source: Adv Sci (Weinh). 2023 Jan 26;10(10):2207693. doi: 10.1002/advs.202207693 (PMC10074045; doi:10.1002/advs.202207693)
Supplement: Supplementary file 1 — Supporting Information [file ADVS-10-2207693-s005.pdf]

ADVANCED SCIENCE
Open Access

Supporting Information

for *Adv. Sci.*, DOI 10.1002/advs.202207693

Transferrin Receptor-Mediated Iron Uptake Promotes Colon Tumorigenesis

*Hyeoncheol Kim, Luke B Villareal, Zhaoli Liu, Mohammad Haneef, Daniel M Falcon, David R Martin, Ho-Joon Lee, Michael K Dame, Durga Attili, Ying Chen, James Varani, Jason R. Spence, Olga Kovbasnjuk, Justin A Colacino, Costas A. Lyssiotis, Henry C Lin, Yatrik M Shah and Xiang Xue**

Supplementary Materials:

Detailed Methods

Supplementary Figures

Supplemental Table 1 DFO modulated genes.

Supplemental Table 2 DAVID_KEGG Pathway.

Supplemental Table 3 DAVID_Cell cycle.

Supplemental Table 4 DAVID_ DNA Replication.

Detailed Methods

Histology analysis, immunofluorescence (IF) and 3,3'-Diaminobenzidine (DAB) enhanced Perl's iron staining

Paraffin sections were deparaffinized and rehydrated with distilled water. For hematoxylin and eosin (H & E) staining, the sections were incubated with hematoxylin solution for 2 minutes and then were washed with tap water for 5 minutes. After washing, the slides were submerged in bluing solution with 1-2 dips. The slides were rinsed with tap water for 2 minutes and incubated with Eosin solution for 5 minutes. After incubation, the sections were dehydrated with 95%, 100% Ethanol and xylene, and covered for pathological examination by a pathologist. For colon injury, Grade 0, no injury; Grade 1, minimal injury; Grade 2, mild injury; Grade 3, moderate injury. For tumorigenesis model, we classified tissues as tubular adenoma with low grade (LG, score 1) versus high grade (HG, score 2) dysplasia, and the involved lesion percentage was estimated. The final pathological score was achieved by multiplying the lesion percentage with the grade score. For IF staining, the sections were put into 10mM sodium citrate buffer at a sub-boiling temperature for 12 minutes. The slides were left on bench top for 2h to cool down, and then were blocked with 10% normal goat serum (NGS) for 1h. Primary antibodies were incubated overnight at 4 Celsius degrees and secondary antibodies were incubated 1h at room temperature. The sections were mounted with EverBrite™ mounting medium (Biotium, Fremont, CA). For DAB enhanced Perl's iron staining, the sections were incubated in a mixture of 2% hydrochloric acid and 1% ferrocyanide solution (1:1) for 30 minutes. After incubation, the sections were rinsed with tap water for 5 minutes. Then, the slides were immersed in 0.05% DAB solution for 20 minutes. The slides were washed with tap water for 5 minutes and then sealed with coverslip using Permount mounting medium (Fisher Scientific, Hampton, NH). Primary antibodies for TFRC (#13113), cleaved Caspase3 (CC3, #9664) and Ki67 (#12202) were purchased from Cell Signaling Technology (Danvers, MA), and γH2AX (sc-517348) was purchased from Santa Cruz Biotechnology (Dallas, TX).

Ferrous iron beads pull down assay

The method used for precipitating proteins by ferrous iron beads was described previously [8]. Briefly, metal-free beads or beads with ferrous iron immobilized onto a pentadentate chelator coupled to a quartz base matrix (PDCSLQ free and Fe-PDC-SLQ) were obtained from Affiland (Liege, Belgium). The beads were washed three times with a volume of EDTA-free Triton lysis buffer (25 mM HEPES, 100 mM NaCl, 10% glycerol, and 1% Triton X-100) twice the volume of the bead bed each time to remove excess unbound ferrous iron. The beads were then used to precipitate proteins from HCT116. Thirty microliters of beads were added to whole-cell lysates (1 mg) in the Triton lysis buffer and rotated at 4ºC for 2 hr. The beads were then washed three times with Triton lysis buffer, pelleted, and the precipitate was resuspended in 5x loading buffer for immunoblotting.

FerroOrange staining

Cells (2×10^5 cells/well) were plated into 24 well plates. After seeding, pre-warmed DMEM containing ferroOrange (0.5 μM, dojindo, Rockville, MD), was incubated at 37 °C for 30 min. After incubation, cell images were taken. Representative images were taken using the RFP channel of an Invitrogen™ EVOS™ FL Auto Imaging System (Thermo Fisher Scientific, Waltham, MA). Fluorescence intensity was quantified using a SpectraMax M2 Microplate Reader (Molecular Devices, Radnor, PA) at excitation 543nm and emission 580nm. The intensity was normalized by protein concentrations.

Transient transfection and luciferase assay.

For luciferase assay, cells were seeded into a 24-well plate at a cell density of 5×10^4 cells per well. TOPflash is a luciferase reporter that contains a minimal fos promoter coupled to Tcf-binding sites upstream of a modified firefly luciferase gene, thus TOPflash yields expression

from the minimal fos promoter and the Wnt/ beta-Catenin activity-sensitive Tcf sites. TOPflash luciferase constructs were co-transfected with pcDNA3 beta-Catenin S33Y (19286, Addgene), TNKS (Homo sapiens) in pLenti6.3/V5-DEST (HsCD00946323, DNASU) by polyethylenimine (PEI; Polysciences Inc., Warrington, PA). HA-E2F-1 wt-pRcCMV (#21667, Addgene), POLD1_pLX307 (#98358, Addgene), pCDH-puro-cMyc (#46970, Addgene) or empty plasmids were transfected into cells using lipofectamine 2000 (Thermo Fisher Scientific). Experiments were carried out 24-48 hours post-transfection. Similarly, SW480 were transfected with c-Myc siRNA (EHU021051), E2F1 siRNA (EHU070981), TNKS siRNA (EHU142711), and AXIN2 siRNA (EHU001481) from Millipore (Burlington, MA).

Quantitative polymerase chain reaction (qPCR) analysis

Total RNA was extracted using IBI Isolate DNA/RNA Reagent Kit (IB47602, IBI Scientific, Dubuque, IA). qPCR was performed using a LightCycler 480 instrument (Roche Diagnostics, Indianapolis, IN). For DSS treated tissue, RNA was further precipitated with 8M LiCl (1/3 volume of RNA solution) and purified. The used pre-designed primers are listed below:

18S_forward, GTAACCCGTTGAACCCCATT

18S_ reverse, CCATCCAATCGGTAGTAGCG

hTFRC forward, ACCATTGTCATATACCCGGTTCA

hTFRC reverse, CAATAGCCCAAGTAGCCAATCAT

mTFRC forward, TCA AGC CAG ATC AGC AATT CTC

mTFRC reverse, AGC CAG TTT CAT CTC CAC ATG

hPOLD1 forward, ATCCAGAACTTCGACCTTCCG

hPOLD1 reverse, ACGGCATTGAGCGTGTAGG

RNA-seq and data analysis.

RNA sequencing libraries were prepared using the TruSeq RNA library prep kit v2 (Illumina) following the manufacturer's recommended protocol. The libraries were sequenced using single-end 50-cycle reads on a HiSeq 4000 sequencer (Illumina) at the University of Michigan DNA Sequencing Core Facility. Raw sequencing read quality was assessed utilizing FastQC. Reads were aligned to the reference human transcriptome (UCSC) using STAR 2.5.2a. Default parameters were used for the alignment, with the exception of "--outFilterMultimapNmax 10" and "--sjdbScore 2". Expression quantification and differential expression analysis between DFO and control tumor colonoids were conducted using CuffDiff v 2.1.1 using default settings. For the CuffDiff analysis, we used Genome Reference Consortium Human Build 37 (GRCh37) as the reference genome. Genes were considered differentially expressed between conditions at a false-discovery rate-adjusted p value of less than 0.05. Differentially expressed pathways were identified utilizing The Database for Annotation, Visualization and Integrated Discovery (DAVID, https://david.ncifcrf.gov/home.jsp). KEGG biological pathways and gene ontology biological processes were considered differentially expressed at a p value of less than 0.05.

Thiazolyl Blue Tetrazolium Bromide (MTT) assay

Cells were plated at a concentration of 5×10^4 cells/mL in 24-well plates. MTT (125μL, 5mg/mL, Sigma, MO) solution was added to each plate and incubated for 30 min. Dimethyl sulfoxide (DMSO) was added and absorbance was measured at 570nm using a BioTek Synergy HTX Multi-Mode Microplate Reader.

Tissue iron assay

Xenograft tumor tissue samples were homogenized in Millipore water (100μL/10mg tissue). Lysates were mixed with an equal volume of acid solution (1M hydrochloric acid, 10% trichloroacetic acid, 10g/L ascorbic acid). Then, the mixture was incubated for 1 hour at 95 degrees. After centrifuge, supernatants were collected and mixed with ferrozine solution (0.5mM

ferrozine, 1.5M sodium acetate and 0.01% mercaptoacetic acid). Absorbance was measured at 562nm to determine tissue iron levels.

Crystal violet staining

Cells ($1x10^3$ cells per well) were seeded in 6-well plates. After 1 week, the cells were washed with PBS once and fixed in 10% formaldehyde for 10 minutes. Then, the cells were stained with 0.05% crystal violet solution for 30 minutes. After incubation, the cells were washed with tap water twice and dried for 5 minutes. Methanol was added to the plate to solubilize crystal violet in the stained cells. Absorbance was measured at 540nm.

Supplementary Figures

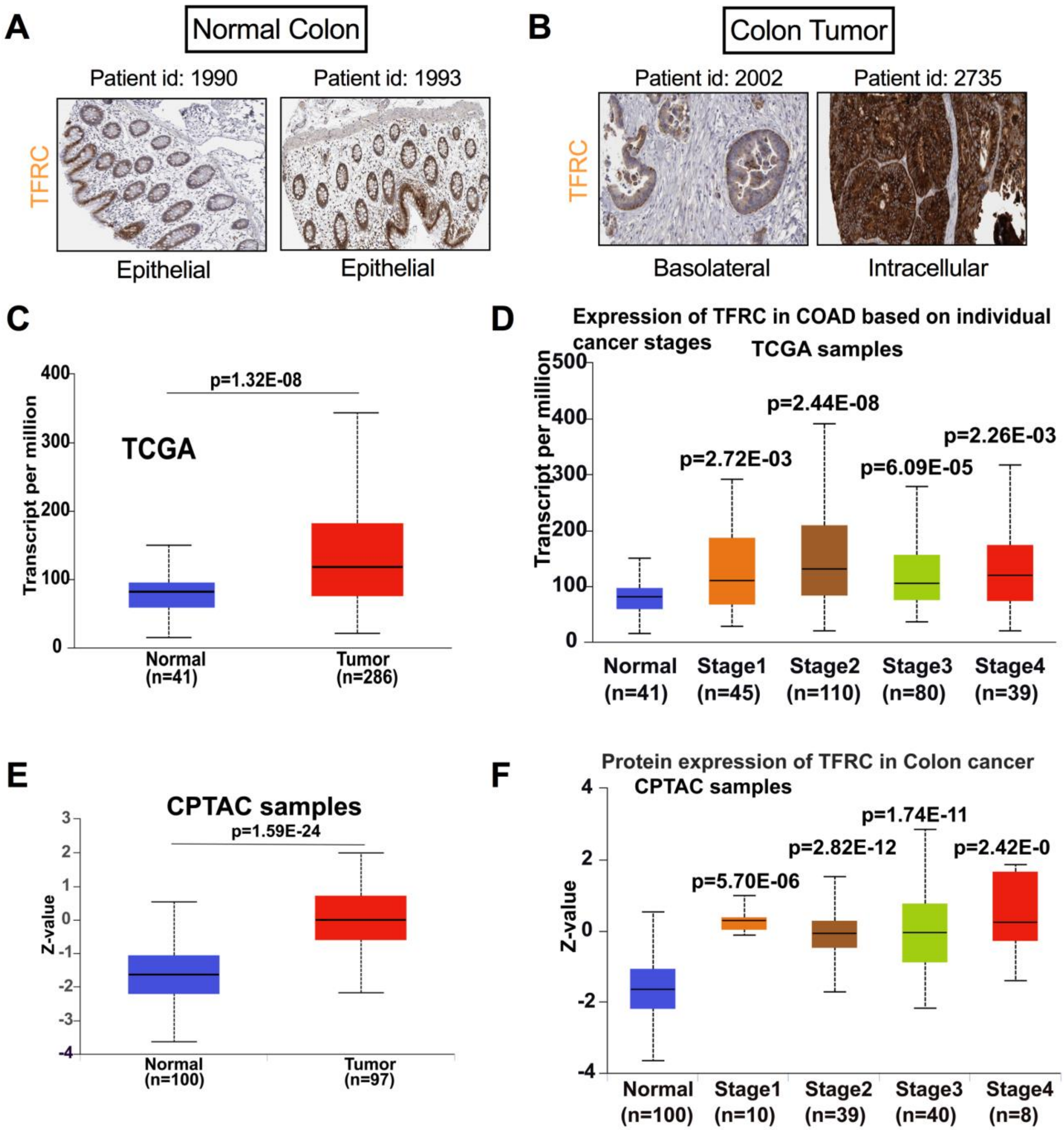

Fig. S1 Cellular and intracellular localization of TFRC in human normal colon and tumor tissues. (**A**) Representative immunohistochemical staining shows epithelial localization of TFRC in normal human colon tissues. (**B**) Representative immunohistochemical staining shows basolateral and intracellular localization of TFRC in human colon tumors. Image credit: Human Protein Atlas. Gene expression of TFRC **(C)** in normal and colon tumors, and **(D)** based on individual cancer stages from TCGA database. Protein expression of TFRC **(E)** in normal and colon tumors, and **(F)** based on individual cancer stages from CPTAC database.

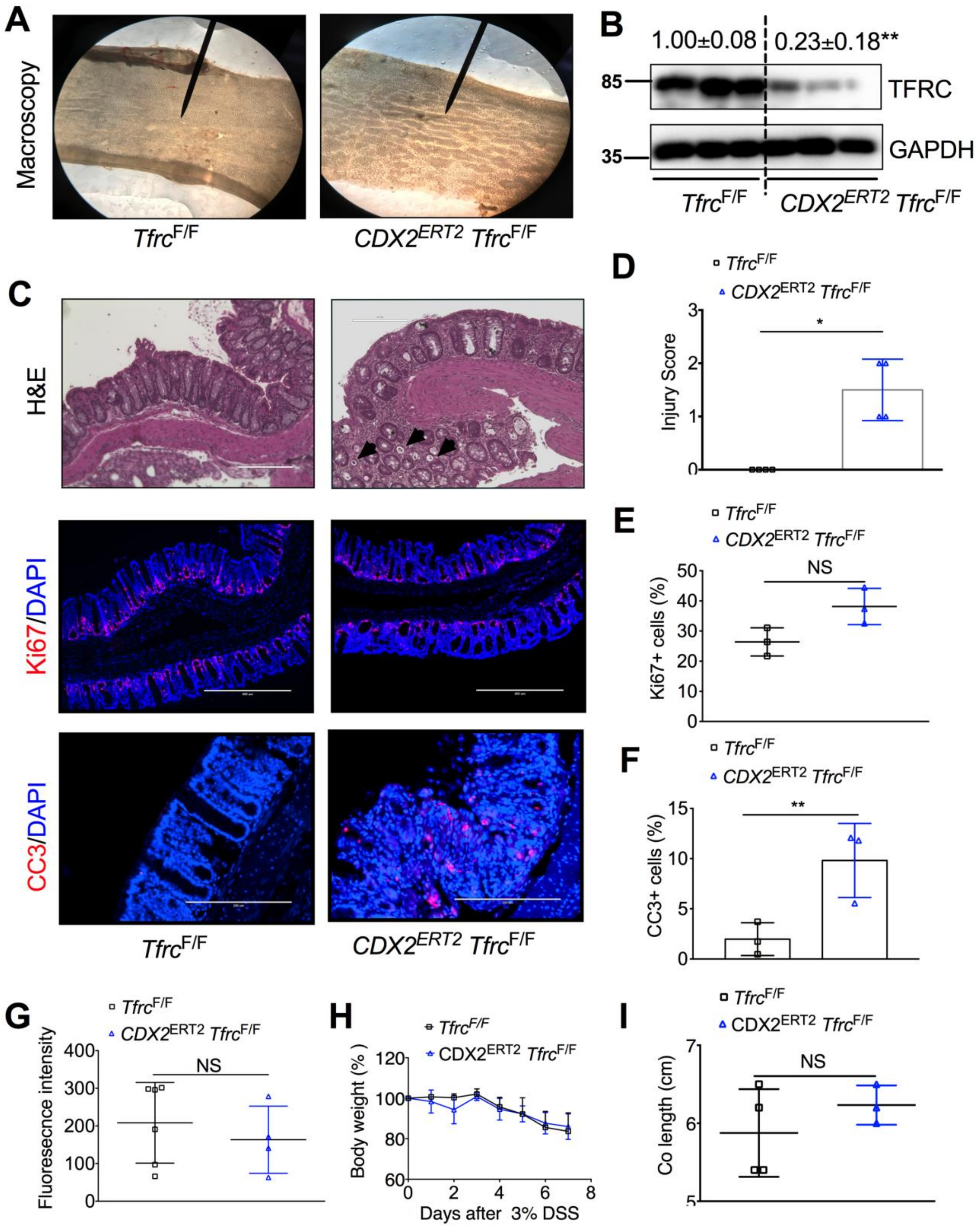
A
Macroscopy
TfrcF/F
CDX2ERT2 TfrcF/F
B
1.00±0.08
0.23±0.18**
85
35
TFRC
GAPDH
TfrcF/F
CDX2ERT2 TfrcF/F
C
H&E
Ki67/DAPI
CC3/DAPI
TfrcF/F
CDX2ERT2 TfrcF/F
D
TfrcF/F
CDX2ERT2 TfrcF/F
Injury Score
*
E
TfrcF/F
CDX2ERT2 TfrcF/F
NS
Ki67+ cells (%)
F
TfrcF/F
CDX2ERT2 TfrcF/F
**
CC3+ cells (%)
G
TfrcF/F
CDX2ERT2 TfrcF/F
NS
Fluoresecnce intensity
H
TfrcF/F
CDX2ERT2 TfrcF/F
Body weight (%)
Days after 3% DSS
I
TfrcF/F
CDX2ERT2 TfrcF/F
NS
Co length (cm)

Fig.S2 TFRC disruption causes colonic injury but does not increase susceptibility to acute colitis. (**A**) Macroscopic images, (**B**) immunoblotting analysis, (**C**) histological staining images, (**D**) histological injury score, and quantification of (**E**) Ki67 staining and (**F**) CC3 staining in colons from *CDX2*^{ERT2} *Tfrc*$^{F/F}$ and *Tfrc*$^{F/F}$ mice (n=3-4) treated with TAM (100mg/kg) for 3 days. Arrowheads indicate tissue injury. (**G**) FITC-dextran fluorescence intensity from serum of *CDX2*^{ERT2} *Tfrc*$^{F/F}$ mice (n=4) and *Tfrc*$^{F/F}$ mice (n=6) treated with 100mg/kg TAM for 3 days. (**H**) Body weight changes and (**I**) colon lengths of *CDX2*^{ERT2} *Tfrc*$^{F/F}$ mice (n=3) and *Tfrc*$^{F/F}$ mice (n=4) treated with TAM (100mg/kg) for 3 days and DSS (3%) for 7 days. *$p<0.05$ and **$p<0.01$. NS, not significant.

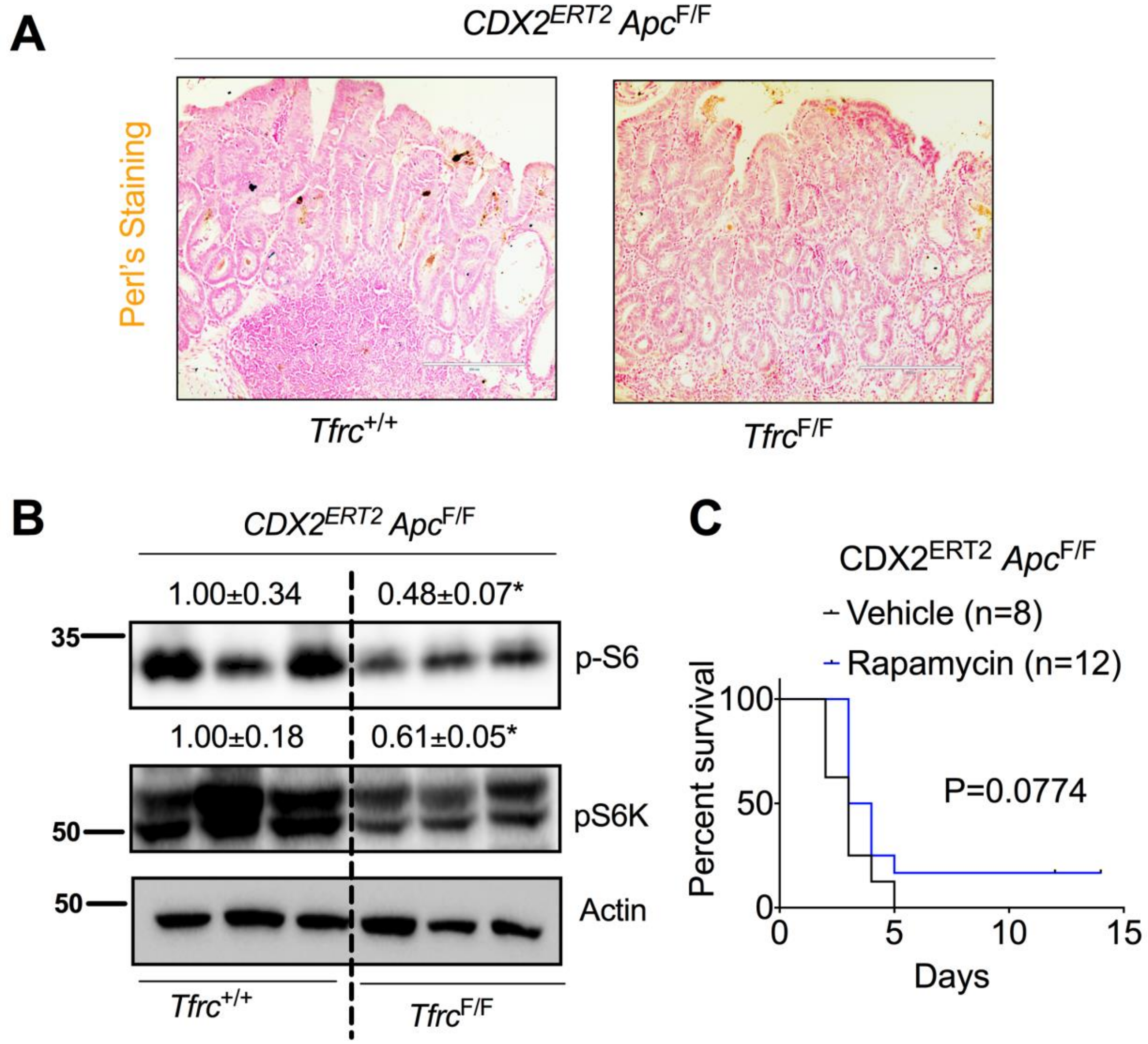

Fig. S3 Rapamycin didn't prolong mouse survival caused by bi-allelic *Apc* loss. (A) DAB-enhanced Perl's iron staining (brown staining indicates iron deposition), (**B**) immunoblotting analysis in *CDX2*^{ERT2} *Tfrc*$^{F/F}$*Apc*$^{F/F}$ mice (n=3) and *CDX2*^{ERT2} *Tfrc*$^{+/+}$*Apc*$^{F/F}$ mice (n=3) treated with TAM (100mg/kg) for 3 days and sacked 7 days after last TAM treatment. **(C)** Survival curve in *CDX2*^{ERT2} *Apc*$^{F/F}$ mice treated with TAM (100mg/kg) for 3 days and DSS (1.5%) for 7 days. Daily i.p. injection of rapamycin (10mg/kg, n=12) or vehicle (n=8) was performed at one day before DSS treatment. *$p<0.05$.

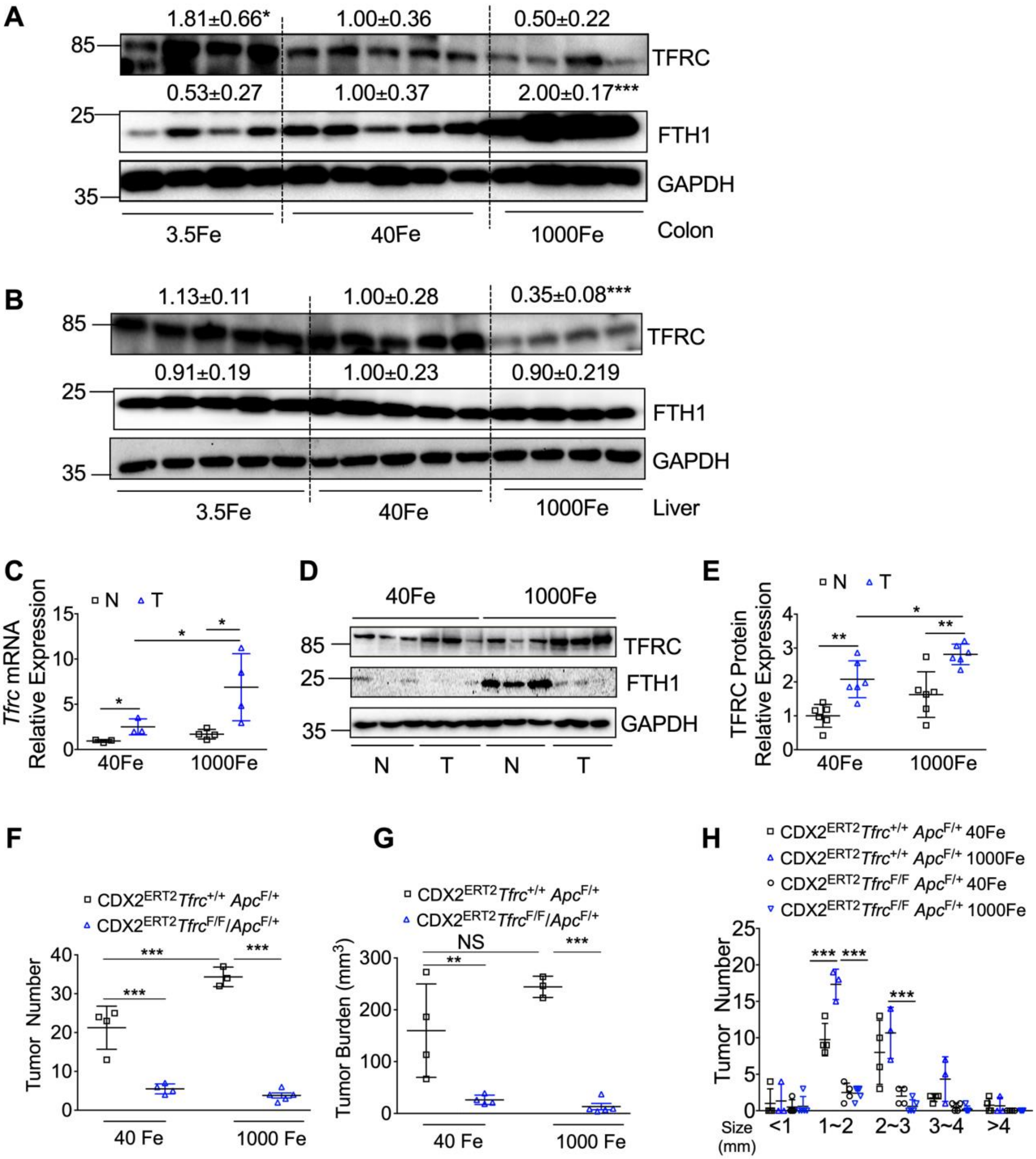

A
1.81±0.66*
1.00±0.36
0.50±0.22
85
TFRC
0.53±0.27
1.00±0.37
2.00±0.17***
25
FTH1
GAPDH
35
3.5Fe
40Fe
1000Fe
Colon
B
1.13±0.11
1.00±0.28
0.35±0.08***
85
TFRC
0.91±0.19
1.00±0.23
0.90±0.219
25
FTH1
GAPDH
35
3.5Fe
40Fe
1000Fe
Liver
C
N T
Tfrc mRNA Relative Expression
40Fe
1000Fe
D
40Fe
1000Fe
TFRC
FTH1
GAPDH
N T N T
E
N T
TFRC Protein Relative Expression
40Fe
1000Fe
F
CDX2ERT2Tfrc+/+ ApcF/+
CDX2ERT2TfrcF/F/ApcF/+
Tumor Number
40 Fe
1000 Fe
G
CDX2ERT2Tfrc+/+ ApcF/+
CDX2ERT2TfrcF/F/ApcF/+
NS
Tumor Burden (mm3)
40 Fe
1000 Fe
H
CDX2ERT2Tfrc+/+ ApcF/+ 40Fe
CDX2ERT2Tfrc+/+ ApcF/+ 1000Fe
CDX2ERT2TfrcF/F ApcF/+ 40Fe
CDX2ERT2TfrcF/F ApcF/+ 1000Fe
Tumor Number
Size (mm)
<1
1~2
2~3
3~4
>4

Fig. S4 Colon-specific TFRC deletion protects mice from high iron-driven colon tumorigenesis. Immunoblotting blot analysis in **(A)** colon or (**B**) liver tissues from C57BL/6 mice (n=4-5) treated with low-iron diet (3.5Fe), iron-replete diet (40Fe) or high-iron diet (1000Fe) for two weeks. *Tfrc* expression by (**C**) qPCR analysis, (**D**) immunoblotting blot analysis and (**E**) quantification in normal (n=3-6) and tumor (n=3-6) colon tissues from $CDX2^{ERT2}$ $Apc^{F/+}$ mice fed with a 40Fe or 1000Fe diet. **(F)** total tumor number, **(G)** tumor burden, and **(H)** tumor number at different sizes in $CDX2^{ERT2}$ $Tfrc^{F/F}Apc^{F/+}$ and $CDX2^{ERT2}$ $Tfrc^{+/+}Apc^{F/+}$ mice treated with TAM (100mg/kg) for 3 days and 2 cycles of DSS (2%) for 7 days with an interval of 14 days regular water. Mice were treated with 40Fe or 1000Fe two days before the initiation of DSS treatment. *p<0.05, **p<0.01, ***p<0.001. NS, not significant.

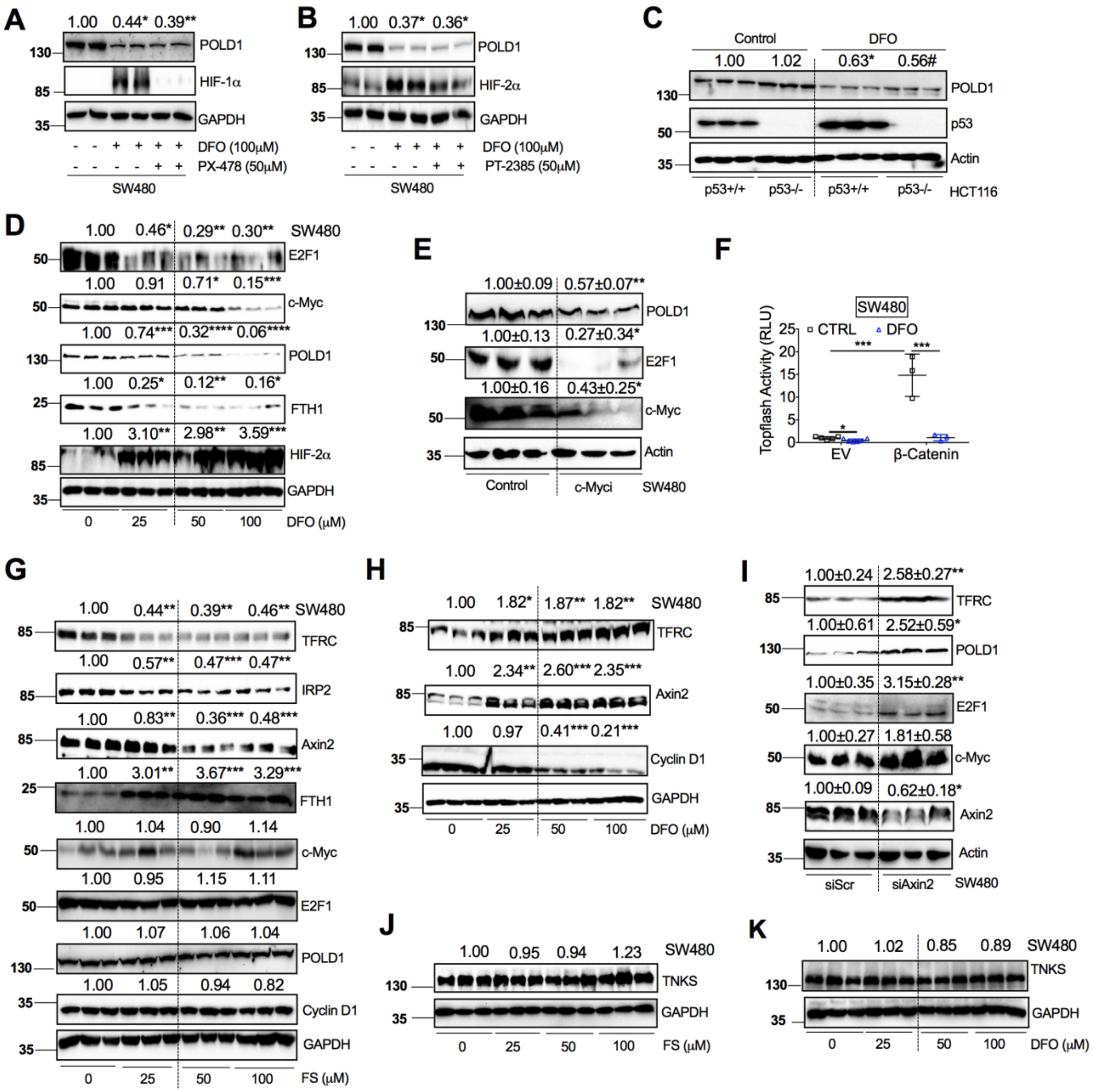

A
1.00 0.44* 0.39**
POLD1
HIF-1α
GAPDH
DFO (100μM)
PX-478 (50μM)
SW480
B
1.00 0.37* 0.36*
POLD1
HIF-2α
GAPDH
DFO (100μM)
PT-2385 (50μM)
SW480
C
Control
DFO
1.00 1.02 0.63* 0.56#
POLD1
p53
Actin
p53+/+ p53-/- p53+/+ p53-/- HCT116
D
1.00 0.46* 0.29** 0.30** SW480
E2F1
1.00 0.91 0.71* 0.15***
c-Myc
1.00 0.74*** 0.32**** 0.06****
POLD1
1.00 0.25* 0.12** 0.16*
FTH1
1.00 3.10** 2.98** 3.59***
HIF-2α
GAPDH
0 25 50 100 DFO (μM)
E
1.00±0.09 0.57±0.07**
POLD1
1.00±0.13 0.27±0.34*
E2F1
1.00±0.16 0.43±0.25*
c-Myc
Actin
Control c-Myci SW480
F
SW480
CTRL DFO
Topflash Activity (RLU)
EV β-Catenin
G
1.00 0.44** 0.39** 0.46** SW480
TFRC
1.00 0.57** 0.47*** 0.47**
IRP2
1.00 0.83** 0.36*** 0.48***
Axin2
1.00 3.01** 3.67*** 3.29***
FTH1
1.00 1.04 0.90 1.14
c-Myc
1.00 0.95 1.15 1.11
E2F1
1.00 1.07 1.06 1.04
POLD1
1.00 1.05 0.94 0.82
Cyclin D1
GAPDH
0 25 50 100 FS (μM)
H
1.00 1.82* 1.87** 1.82** SW480
TFRC
1.00 2.34** 2.60*** 2.35***
Axin2
1.00 0.97 0.41*** 0.21***
Cyclin D1
GAPDH
0 25 50 100 DFO (μM)
I
1.00±0.24 2.58±0.27**
TFRC
1.00±0.61 2.52±0.59*
POLD1
1.00±0.35 3.15±0.28**
E2F1
1.00±0.27 1.81±0.58
c-Myc
1.00±0.09 0.62±0.18*
Axin2
Actin
siScr siAxin2 SW480
J
1.00 0.95 0.94 1.23 SW480
TNKS
GAPDH
0 25 50 100 FS (μM)
K
1.00 1.02 0.85 0.89 SW480
TNKS
GAPDH
0 25 50 100 DFO (μM)

Fig. S5 POLD1 is regulated by E2F1 and binds with iron. Immunoblotting blot analysis in SW480 cells treated with or without (**A**) HIF-1α inhibitor PX-478, or (**B**) HIF-2α inhibitor PT-2385 following DFO treatment. (**C**) Immunoblotting blot analysis in HCT116 p53+/+ and p53-/- cells with or without DFO treatment. **(D)** Immunoblotting blot analysis of SW480 cells treated with different doses of DFO. **(E)** Immunoblotting blot analysis of SW480 cells treated with or without c-Myc inhibitor (c-Myci) 10058-F4 overnight. (**F**) Topflash assay in SW480 transfected with control (EV) or β-catenin followed with or without DFO treatment overnight. (**G**) Immunoblotting blot analysis in SW480 treated with different doses of FS. (**H**) Immunoblotting blot analysis in SW480 treated with different doses of DFO. (**I**) Immunoblotting blot analysis in SW480 transfected with Control (siScr) or AXIN2 siRNA. Immunoblotting blot analysis in SW480 treated with different doses of (**J**) FS or (**K**) DFO. Immunoblotting blot analysis in SW480 transfected with siScr or TNKS siRNA. *$p<0.05$, **$p<0.01$, ***$p<0.001$ and ****$p<0.001$. #$p<0.05$ vs p53-/-.

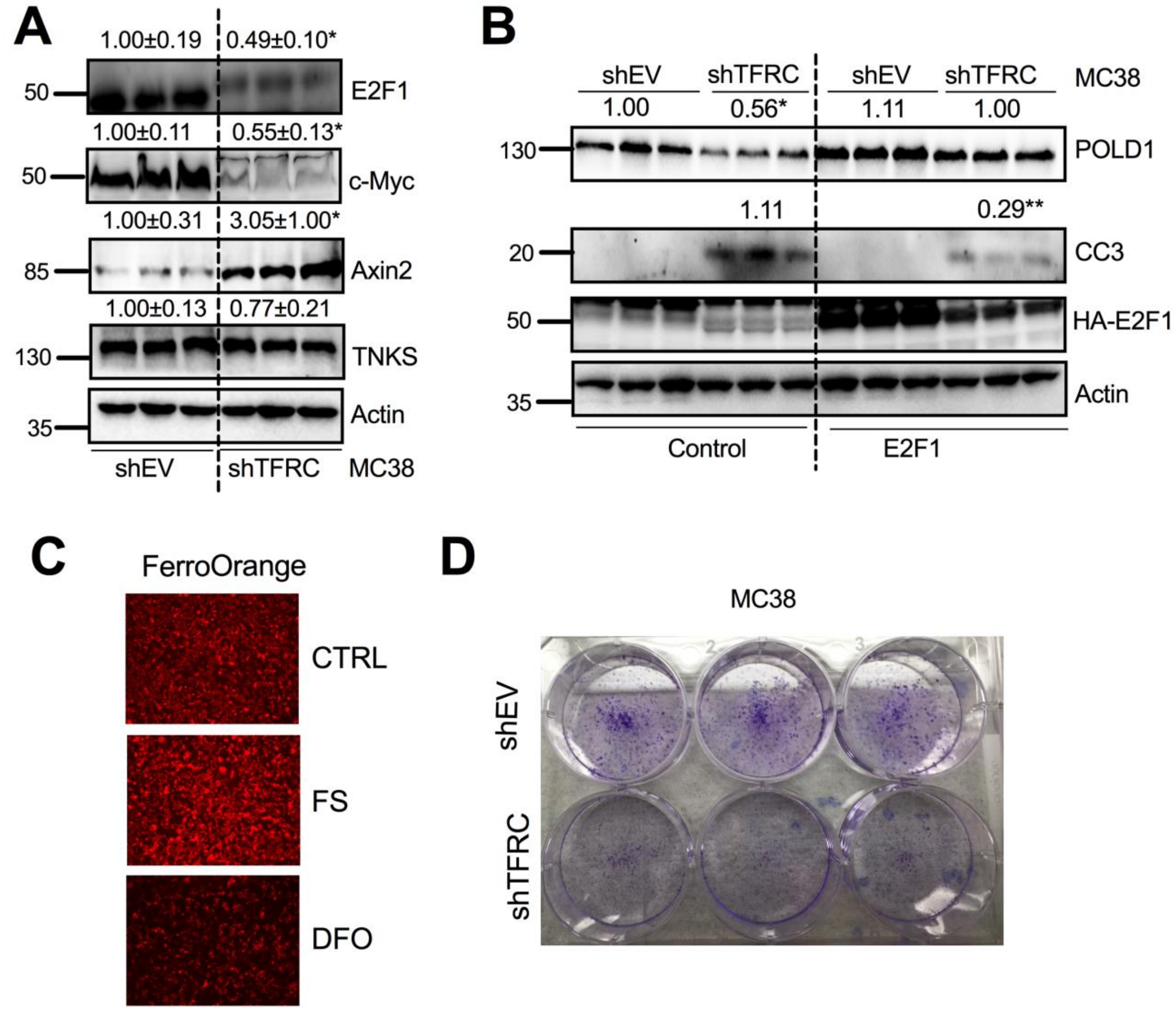

Fig. S6 E2F1 is critical for POLD1 regulation in TFRC knockdown cells. (**A**) Immunoblot analysis of MC38 shTFRC or shEV cells. **(B)** Immunoblotting blot analysis of MC38 shTFRC or shEV cells transfected with HA-E2F1. (**C**) Representative Ferro-Orange staining images of MC38 cell treated with vehicle control (CTRL), FS, or DFO. (**D**) Representative crystal violet staining images for colony formation assay from MC38 cells with or without TFRC knockdown. *p<0.05 and **p<0.01,

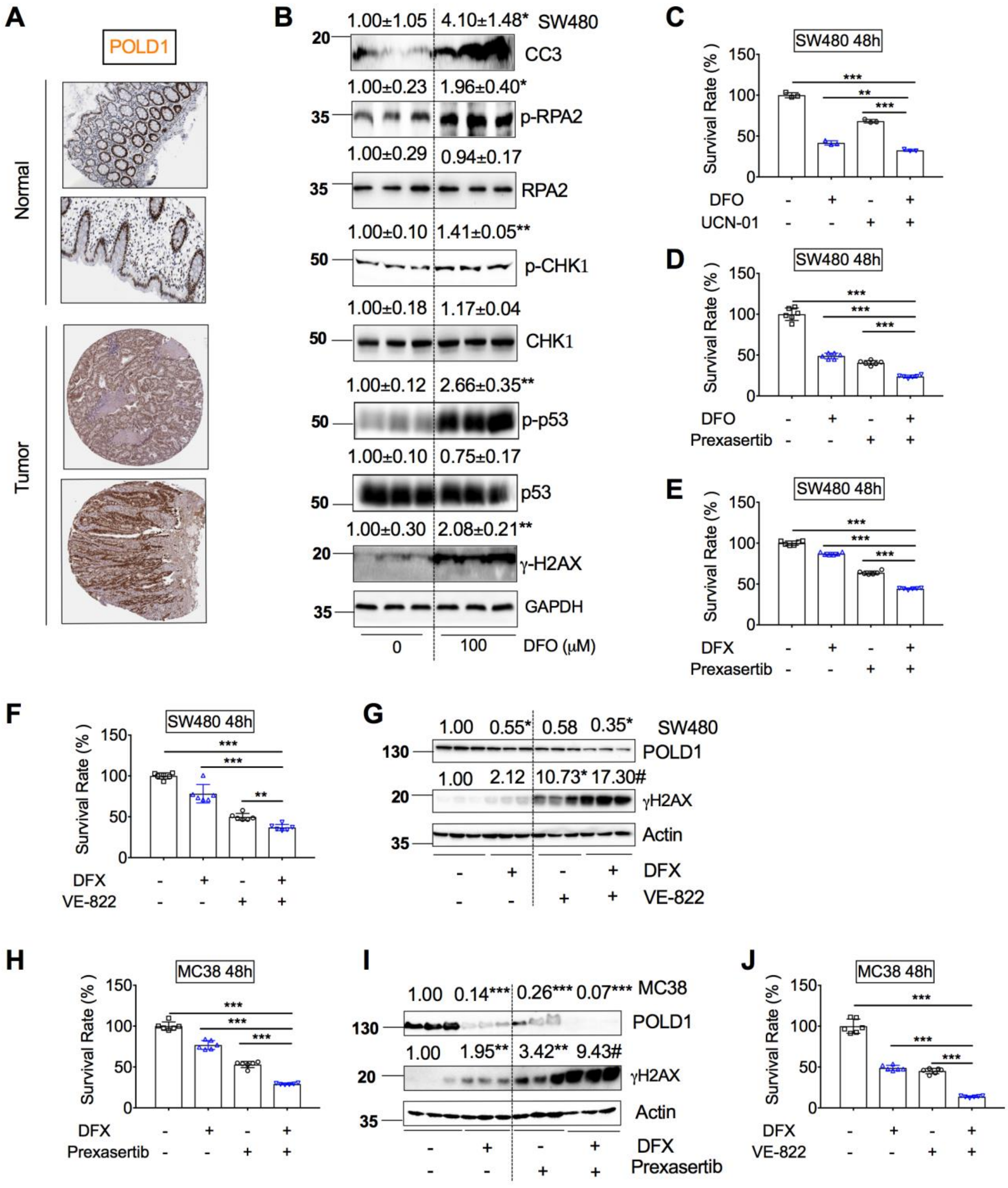
A
POLD1
Normal
Tumor
B
1.00±1.05 4.10±1.48* SW480
CC3
1.00±0.23 1.96±0.40*
p-RPA2
1.00±0.29 0.94±0.17
RPA2
1.00±0.10 1.41±0.05**
p-CHK1
1.00±0.18 1.17±0.04
CHK1
1.00±0.12 2.66±0.35**
p-p53
1.00±0.10 0.75±0.17
p53
1.00±0.30 2.08±0.21**
γ-H2AX
GAPDH
0 100 DFO (μM)
C
SW480 48h
Survival Rate (%)
DFO
UCN-01
D
SW480 48h
DFO
Prexasertib
E
SW480 48h
DFX
Prexasertib
F
SW480 48h
DFX
VE-822
G
1.00 0.55* 0.58 0.35* SW480
POLD1
1.00 2.12 10.73* 17.30#
γH2AX
Actin
DFX
VE-822
H
MC38 48h
DFX
Prexasertib
I
1.00 0.14*** 0.26*** 0.07*** MC38
POLD1
1.00 1.95** 3.42** 9.43#
γH2AX
Actin
DFX
Prexasertib
J
MC38 48h
DFX
VE-822

Fig. S7 Iron-POLD1 axis is critical for DNA damage response and apoptosis. (**A**) Representative immunohistochemical staining shows increased POLD1 expression in human colon tumors compared to normal human colon tissues. Image credit: Human Protein Atlas. **(B)** Immunoblotting blot analysis in colon-derived SW480 cancer cells following DFO (100μM) treatment for overnight. (**C**) MTT assay in SW480 cells treated with DFO (10μM) and/or CHK1 inhibitor UCN-01 (100nM), or (**D**) CHK1 inhibitor, Prexasertib (10μM DFO and/or 100nM) for 48 hours. (**E**) MTT assay in SW480 cells treated with DFX (10μM), and/or Prexasertib (100nM) for 48 hours. (**F**) MTT assay and (**G**) immunoblotting blot analysis in SW480 treated with DFX (10μM) and/or VE-822 (10μM) for 48 hours. (**H**) MTT assay and (**I**) immunoblotting blot analysis in MC38 cells treated with DFX (10μM) and/or Prexasertib (100nM) for 48 hours. (**J**) MTT assay in MC38 cells treated with DFX (10μM) and/or ATR inhibitor, VE-822 (10μM). *p<0.05, **p<0.01 and ***p<0.01. #p<0.05 vs DFX.
