# Supplementary material for: Transferrin Receptor‐Mediated Iron Uptake Promotes Colon Tumorigenesis
Source: Adv Sci (Weinh). 2023 Jan 26;10(10):2207693. doi: 10.1002/advs.202207693 (PMC10074045; doi:10.1002/advs.202207693)
Supplement: Supplementary file 3 — Supplemental TableS2 [file ADVS-10-2207693-s003.pdf]

ADVANCED SCIENCE
Open Access

Supporting Information

for *Adv. Sci.*, DOI 10.1002/advs.202207693

Transferrin Receptor-Mediated Iron Uptake Promotes Colon Tumorigenesis

*Hyeoncheol Kim, Luke B Villareal, Zhaoli Liu, Mohammad Haneef, Daniel M Falcon, David R Martin, Ho-Joon Lee, Michael K Dame, Durga Attili, Ying Chen, James Varani, Jason R. Spence, Olga Kovbasnjuk, Justin A Colacino, Costas A. Lyssiotis, Henry C Lin, Yatrik M Shah and Xiang Xue**

Functional Annotation Chart

Help and Manual

Current Gene List: List_1
Current Background: Homo sapiens
783 DAVID IDs
⊞ **Options**

Rerun Using Options | Create Sublist

28 chart records

Download File

Sublist	Category	Term	RT	Genes	Count	%	P-Value	Benjamini
☐	KEGG_PATHWAY	Cell cycle	RT		33	4.2	7.9E-16	1.8E-13
☐	KEGG_PATHWAY	DNA replication	RT		16	2.0	2.1E-11	2.5E-9
☐	KEGG_PATHWAY	HTLV-I infection	RT		30	3.8	7.7E-6	6.0E-4
☐	KEGG_PATHWAY	Pathways in cancer	RT		38	4.9	3.7E-5	2.2E-3
☐	KEGG_PATHWAY	Mismatch repair	RT		7	0.9	5.4E-4	2.3E-2
☐	KEGG_PATHWAY	Small cell lung cancer	RT		13	1.7	5.9E-4	2.3E-2
☐	KEGG_PATHWAY	Base excision repair	RT		8	1.0	7.3E-4	2.4E-2
☐	KEGG_PATHWAY	p53 signaling pathway	RT		11	1.4	1.1E-3	3.2E-2
☐	KEGG_PATHWAY	ECM-receptor interaction	RT		11	1.4	7.6E-3	1.9E-1
☐	KEGG_PATHWAY	Viral carcinogenesis	RT		19	2.4	8.0E-3	1.9E-1
☐	KEGG_PATHWAY	MAPK signaling pathway	RT		21	2.7	1.6E-2	3.4E-1
☐	KEGG_PATHWAY	Pancreatic cancer	RT		8	1.0	3.3E-2	5.7E-1
☐	KEGG_PATHWAY	Renal cell carcinoma	RT		8	1.0	3.5E-2	5.7E-1
☐	KEGG_PATHWAY	PI3K-Akt signaling pathway	RT		25	3.2	3.6E-2	5.7E-1
☐	KEGG_PATHWAY	Oocyte meiosis	RT		11	1.4	3.6E-2	5.7E-1
☐	KEGG_PATHWAY	Bile secretion	RT		8	1.0	4.3E-2	6.2E-1
☐	KEGG_PATHWAY	Osteoclast differentiation	RT		12	1.5	4.5E-2	6.2E-1
☐	KEGG_PATHWAY	FoxO signaling pathway	RT		12	1.5	5.2E-2	6.4E-1
☐	KEGG_PATHWAY	Hematopoietic cell lineage	RT		9	1.1	5.2E-2	6.4E-1
☐	KEGG_PATHWAY	Mineral absorption	RT		6	0.8	5.5E-2	6.4E-1
☐	KEGG_PATHWAY	Influenza A	RT		14	1.8	6.8E-2	7.3E-1
☐	KEGG_PATHWAY	Nucleotide excision repair	RT		6	0.8	6.9E-2	7.3E-1
☐	KEGG_PATHWAY	Axon guidance	RT		11	1.4	7.7E-2	7.3E-1
☐	KEGG_PATHWAY	Retinol metabolism	RT		7	0.9	8.0E-2	7.3E-1
☐	KEGG_PATHWAY	Inflammatory bowel disease (IBD)	RT		7	0.9	8.0E-2	7.3E-1
☐	KEGG_PATHWAY	Hepatitis B	RT		12	1.5	8.1E-2	7.3E-1
☐	KEGG_PATHWAY	Glycolysis / Gluconeogenesis	RT		7	0.9	9.5E-2	8.1E-1
☐	KEGG_PATHWAY	Hepatitis C	RT		11	1.4	9.8E-2	8.1E-1

603 gene(s) from your list are not in the output.
