# Supplementary material for: Transferrin Receptor‐Mediated Iron Uptake Promotes Colon Tumorigenesis
Source: Adv Sci (Weinh). 2023 Jan 26;10(10):2207693. doi: 10.1002/advs.202207693 (PMC10074045; doi:10.1002/advs.202207693)
Supplement: Supplementary file 4 — Supplemental TableS3 [file ADVS-10-2207693-s004.pdf]

ADVANCED SCIENCE
Open Access

Supporting Information

for *Adv. Sci.*, DOI 10.1002/advs.202207693

Transferrin Receptor-Mediated Iron Uptake Promotes Colon Tumorigenesis

*Hyeoncheol Kim, Luke B Villareal, Zhaoli Liu, Mohammad Haneef, Daniel M Falcon, David R Martin, Ho-Joon Lee, Michael K Dame, Durga Attili, Ying Chen, James Varani, Jason R. Spence, Olga Kovbasnjuk, Justin A Colacino, Costas A. Lyssiotis, Henry C Lin, Yatrik M Shah and Xiang Xue**

National Institute of Allergy and Infectious Disease
The Database for Annotation, Visualization and Integrated Discovery

Gene Report

Help and Manual

Current Gene List: List_1
Current Background: Homo sapiens
783 DAVID IDs
33 record(s)

Download File

OFFICIAL_GENE_SYMBOL	GENE NAME	Related Genes	Species
BUB1B	BUB1 mitotic checkpoint serine/threonine kinase B(BUB1B)	RG	Homo sapiens
BUB1	BUB1 mitotic checkpoint serine/threonine kinase(BUB1)	RG	Homo sapiens
E2F1	E2F transcription factor 1(E2F1)	RG	Homo sapiens
E2F2	E2F transcription factor 2(E2F2)	RG	Homo sapiens
MAD2L1	MAD2 mitotic arrest deficient-like 1 (yeast)(MAD2L1)	RG	Homo sapiens
RBL1	RB transcriptional corepressor like 1(RBL1)	RG	Homo sapiens
SKP2	S-phase kinase associated protein 2(SKP2)	RG	Homo sapiens
SMAD3	SMAD family member 3(SMAD3)	RG	Homo sapiens
TTK	TTK protein kinase(TTK)	RG	Homo sapiens
CDC20	cell division cycle 20(CDC20)	RG	Homo sapiens
CDC25A	cell division cycle 25A(CDC25A)	RG	Homo sapiens
CDC45	cell division cycle 45(CDC45)	RG	Homo sapiens
CDC7	cell division cycle 7(CDC7)	RG	Homo sapiens
CHEK1	checkpoint kinase 1(CHEK1)	RG	Homo sapiens
CCNB1	cyclin B1(CCNB1)	RG	Homo sapiens
CCNB2	cyclin B2(CCNB2)	RG	Homo sapiens
CDK1	cyclin dependent kinase 1(CDK1)	RG	Homo sapiens
CDK4	cyclin dependent kinase 4(CDK4)	RG	Homo sapiens
CDKN2B	cyclin dependent kinase inhibitor 2B(CDKN2B)	RG	Homo sapiens
CDKN2C	cyclin dependent kinase inhibitor 2C(CDKN2C)	RG	Homo sapiens
ESPL1	extra spindle pole bodies like 1, separase(ESPL1)	RG	Homo sapiens
GADD45B	growth arrest and DNA damage inducible beta(GADD45B)	RG	Homo sapiens
MCM2	minichromosome maintenance complex component 2(MCM2)	RG	Homo sapiens
MCM3	minichromosome maintenance complex component 3(MCM3)	RG	Homo sapiens
MCM4	minichromosome maintenance complex component 4(MCM4)	RG	Homo sapiens
MCM5	minichromosome maintenance complex component 5(MCM5)	RG	Homo sapiens
MCM6	minichromosome maintenance complex component 6(MCM6)	RG	Homo sapiens
MCM7	minichromosome maintenance complex component 7(MCM7)	RG	Homo sapiens
ORC1	origin recognition complex subunit 1(ORC1)	RG	Homo sapiens
PTTG1	pituitary tumor-transforming 1(PTTG1)	RG	Homo sapiens
PCNA	proliferating cell nuclear antigen(PCNA)	RG	Homo sapiens
SFN	stratifin(SFN)	RG	Homo sapiens
TGFB1	transforming growth factor beta 1(TGFB1)	RG	Homo sapiens
