# Supplementary material for: Transferrin Receptor‐Mediated Iron Uptake Promotes Colon Tumorigenesis
Source: Adv Sci (Weinh). 2023 Jan 26;10(10):2207693. doi: 10.1002/advs.202207693 (PMC10074045; doi:10.1002/advs.202207693)
Supplement: Supplementary file 5 — Supplemental TableS4 [file ADVS-10-2207693-s002.pdf]

ADVANCED SCIENCE
Open Access

Supporting Information

for *Adv. Sci.*, DOI 10.1002/advs.202207693

Transferrin Receptor-Mediated Iron Uptake Promotes Colon Tumorigenesis

*Hyeoncheol Kim, Luke B Villareal, Zhaoli Liu, Mohammad Haneef, Daniel M Falcon, David R Martin, Ho-Joon Lee, Michael K Dame, Durga Attili, Ying Chen, James Varani, Jason R. Spence, Olga Kovbasnjuk, Justin A Colacino, Costas A. Lyssiotis, Henry C Lin, Yatrik M Shah and Xiang Xue**

National Institute of Allergy and Infectious Disease
The Database for Annotation, Visualization and Integrated Discovery

Gene Report

Help and Manual

Current Gene List: List_1
Current Background: Homo sapiens
783 DAVID IDs
16 record(s)

Download File

OFFICIAL_GENE_SYMBOL	GENE NAME	Related Genes	Species
LIG1	DNA ligase 1(LIG1)	RG	Homo sapiens
POLA1	DNA polymerase alpha 1, catalytic subunit(POLA1)	RG	Homo sapiens
POLD1	DNA polymerase delta 1, catalytic subunit(POLD1)	RG	Homo sapiens
POLE2	DNA polymerase epsilon 2, accessory subunit(POLE2)	RG	Homo sapiens
DNA2	DNA replication helicase/nuclease 2(DNA2)	RG	Homo sapiens
FEN1	flap structure-specific endonuclease 1(FEN1)	RG	Homo sapiens
MCM2	minichromosome maintenance complex component 2(MCM2)	RG	Homo sapiens
MCM3	minichromosome maintenance complex component 3(MCM3)	RG	Homo sapiens
MCM4	minichromosome maintenance complex component 4(MCM4)	RG	Homo sapiens
MCM5	minichromosome maintenance complex component 5(MCM5)	RG	Homo sapiens
MCM6	minichromosome maintenance complex component 6(MCM6)	RG	Homo sapiens
MCM7	minichromosome maintenance complex component 7(MCM7)	RG	Homo sapiens
PRIM1	primase (DNA) subunit 1(PRIM1)	RG	Homo sapiens
PCNA	proliferating cell nuclear antigen(PCNA)	RG	Homo sapiens
RFC3	replication factor C subunit 3(RFC3)	RG	Homo sapiens
RFC5	replication factor C subunit 5(RFC5)	RG	Homo sapiens
